# Single-cell atlas of the mouse ovary reveals molecular drivers of aging and senescence during the estropausal transition

**DOI:** 10.1073/pnas.2600323123

**Published:** 2026-07-21

**Authors:** Xifan Wang, Jiping Yang, Chen Jin, Xizhe Wang, Daniela Contreras, Melody Devos, Maggie M. Kane, Michael G. Rosenfeld, Yousin Suh

**Affiliations:** ^a^ Department of Obstetrics and Gynecology, Columbia University Irving Medical Center, New York, NY 10032; ^b^ Department of Genetics and Development, Columbia University Irving Medical Center, New York, NY 10032; ^c^ Department of Medicine, University of California San Diego, La Jolla, CA 92093

**Keywords:** ovary, aging, cellular senescence, estropausal transition, single-cell RNA-seq

## Abstract

Reproductive aging in mice leads to estropause, characterized by estrous cycle irregularity and eventual cessation, yet its underlying mechanism remains unclear. Here, we present a comprehensive single-cell atlas of mouse ovaries across precisely defined reproductive stages—from young (regular cycling) through the estropausal transition (regular vs. irregular cycling) to post-estropause (acyclic)—and of ovary-specific senescent cells defined by high senescence-associated β-galactosidase activity. We mapped transcriptomic dynamics of ovarian aging and characterized the molecular features of ovarian senescent cells. Our analyses revealed that during the estropausal transition, irregularly cycling ovaries exhibited accelerated aging and cellular senescence features compared with regularly cycling counterparts, including increased transcriptional noise, altered conserved aging pathways such as oxidative phosphorylation and proteostasis, hormone dysregulation in granulosa cells, and elevated expression of the senescence marker *Cdkn1a* and senescence-associated secretory phenotype factors. This atlas delineates the cellular and molecular hallmarks of mouse ovarian aging and ovary-specific senescent cells, providing a resource for understanding the mechanisms underlying the estropausal transition.

## Significance

In this study, we present an integrated single-cell view of the molecular and cellular basis of ovarian aging, ovary-specific senescent cells, and their roles in the estropausal transition. By precisely defining reproductive stages and controlling for estrous cycle phase, we captured subtle transcriptomic dynamics of mouse ovarian aging across the reproductive lifespan. We provide a single-cell-level definition of the molecular features of ovary-specific senescent cells with high SA-β-gal activity and identify aging signatures in ovarian somatic cells, particularly granulosa cells, that drive estropausal progression. Our findings fill a critical gap in the field and offer a valuable resource for discovering biomarkers and therapeutic targets to improve ovarian health during aging.

Female reproductive aging impacts not only fertility but also overall health and lifespan (1, 2). In mice, reproductive aging is characterized by progressive disruption of regular estrous cyclicity (normally 4 - 5 d), ultimately culminating in estropause, a process analogous to menopause in women (3–6). Mice typically enter estropausal transition (peri-estropausal) at 9 to 12 mo of age, developing irregular cycles extending to 5 to 8 d, and eventually progress to complete cessation of cyclicity (acyclicity) between 13 to 16 mo, termed post-estropause (4, 7). Even with identical genetic backgrounds and environments, considerable heterogeneity exists in the onset of estropausal transition in mice, mirroring the interindividual variability observed in women’s perimenopausal transition (8).

The ovary is the key organ that maintains female reproductive and endocrine function, and the first organ to undergo profound age-associated functional decline (9). Ovarian aging is marked by a progressive decline in follicle number and oocyte quality (10), accompanied by increased inflammation, collagen deposition, fibrosis, and cellular senescence (11). The aging process of ovary occurs concomitantly with estrous cycle irregularity and cessation, suggesting the intrinsic aging mechanisms of the ovary may drive the estropausal transition. However, a comprehensive understanding of the cellular and molecular alterations occurring within the ovary during aging, and their association with the onset of the estropausal transition, remains lacking.

The ovary encompasses various cell types, including oocytes surrounded by granulosa and theca cells within follicles, alongside supporting somatic cells like stromal, vascular, and immune cells, collectively orchestrating its dynamic functions in follicular development and hormone production. The complexity of ovarian cellular composition makes single-cell RNA sequencing (scRNA-seq) invaluable for revealing cell type–specific alterations in gene expression and intercellular communication during ovarian aging and estropausal transition. Initial scRNA-seq studies have provided preliminary insights into mouse ovarian aging. One study, which examined 3- and 9-mo-old mice, reported increased lymphocyte proportions and stromal fibrosis as potential early markers of ovarian decline (12). Another investigation profiling aging transcriptomes across female reproductive organs showed that fibroblast-driven inflammation and fibrosis drive aging in the uterus and oviduct, but not in the ovary, suggesting that mechanisms underlying ovarian aging remain unclear (13). To achieve a more precise understanding of ovarian aging and the dynamic shifts during the estropausal transition, defining reproductive stages is essential to capture subtle changes and prevent mixing ovaries at different stages, a limitation overlooked in previous studies. Moreover, senescent cells in the ovary remain poorly characterized due to their rarity and heterogeneity (14), highlighting the need for a single-cell atlas to define these populations and identify reliable biomarkers.

In this study, we performed single-cell RNA sequencing on mouse ovaries across precisely defined reproductive stages—from young (regular cycling) through peri-estropause (regular vs. irregular cycling) to post-estropause (acyclic)—as well as on ovarian senescent cells identified by high senescence-associated β-galactosidase activity. We delineated the global and cell type–specific transcriptomic dynamics of ovarian aging and characterized the cellular and molecular features of ovarian senescent cells. By further comparing irregular-cycling ovaries with their regularly cycling counterparts at the same chronological age, we identified molecular drivers underlying the estropausal transition, which is associated with accelerated aging and cellular senescence in the ovary. This study provides a valuable resource for understanding the molecular and cellular mechanisms underlying ovarian aging, ovary-specific senescent cells, and their association with the estropausal transition in mice.

## Results

### Single-Cell Profiling of Mouse Ovaries across Defined Reproductive Stages.

**Fig. 1.**
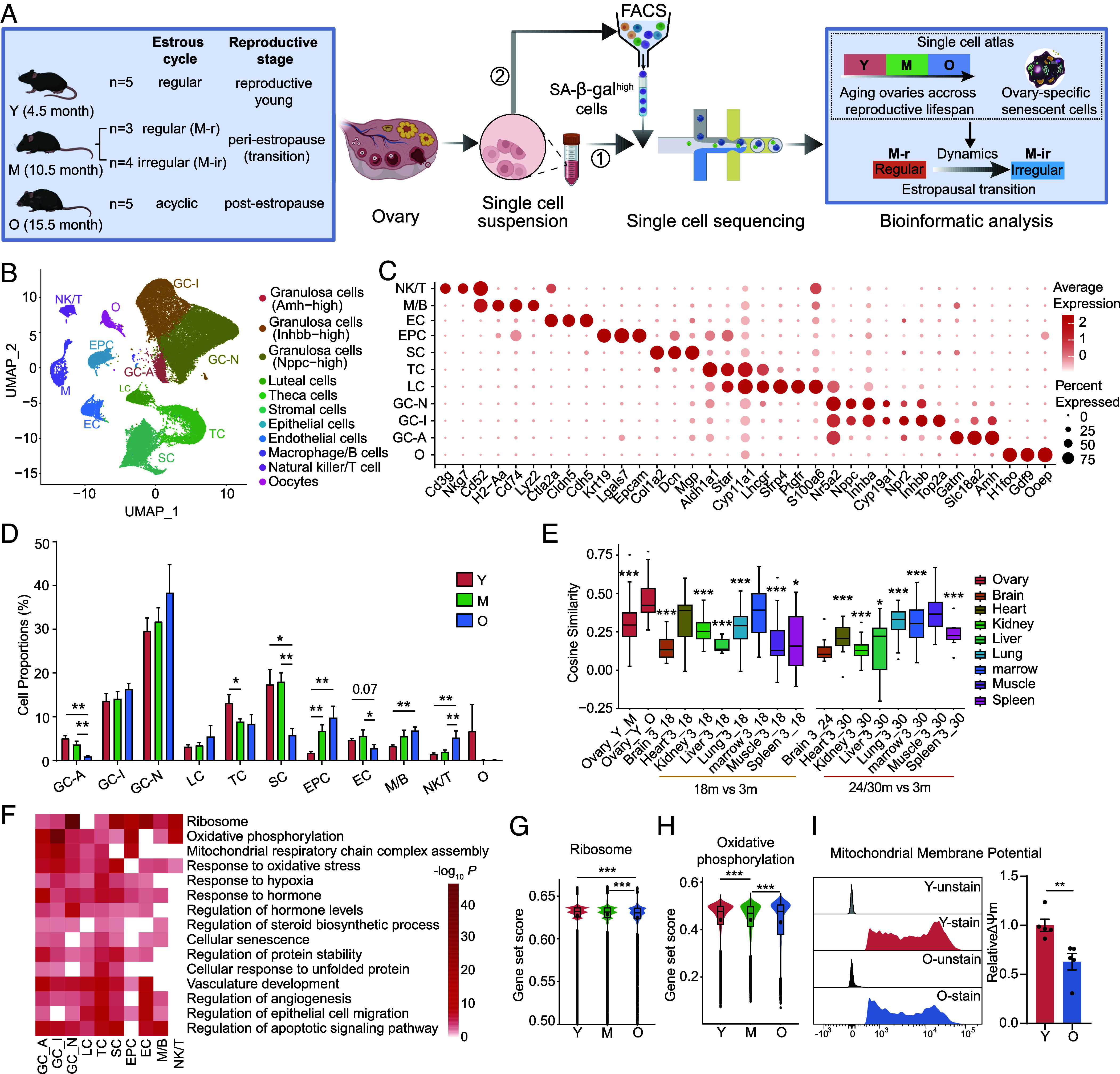
scRNA-seq profiling of mouse ovaries across reproductive aging. (*A*) Flowchart summarizing single-cell RNA sequencing of mouse ovaries from precisely defined reproductive stages and of ovarian senescent cells. FACS, fluorescence-activated cell sorting; SA-β-gal, senescence-associated β-galactosidase activity. (*B*) Uniform manifold approximation and projection (UMAP) plots showing the cell types of mouse ovary. O, oocyte; GC-A, *Amh*-high granulosa cell; GC-I, *Inhbb*-high granulosa cell; GC-N, *Nppc*-high granulosa cell; LC, luteal cell; TC, theca cell; SC, stomal cell; EPC, epithelial cell; EC, endothelial cell; M/B, macrophage/B cell; NK/T, natural killer/T cell. (*C*) Dot plots showing the expression of representative genes for each cell type. (*D*) Bar plots showing the proportion of each cell type in young (Y), peri-estropause (M), and post-estropause (O) ovaries estimated from scRNA-seq data. (Mean ± SEM; Permutation test; *Padj < 0.05, **Padj < 0.01). (*E*) Box plots comparing the cosine similarities of transcriptomic changes during aging between different age groups and tissues (Wilcoxon test; *Padj < 0.05, ***Padj < 0.001). (*F*) Heatmap showing the representative GO terms of monotonic DEGs in each cell type. (*G* and *H*) Violin plots and box plots showing the gene set score of ribosome (*G*) and oxidative phosphorylation (*H*) during mouse ovarian aging. Dot mark indicating the mean. Wilcoxon test; ***Padj < 0.001. (*I*) Flow cytometry analysis of mitochondrial membrane potential in mouse ovarian cells. The *Left* Panel showed representative histograms of mitochondrial membrane potential captured in the PE channel. The *Right* panel showed the statistical comparison of average mitochondrial membrane potential between young (Y) and post-estropause (O) groups. Mitochondrial membrane potential strengths were normalized to the young group. (Mean ± SEM; *t* test; **P < 0.01; n = 5).

To dissect the temporal dynamic signature of mouse ovarian aging across reproductive lifespan, we conducted single-cell RNA sequencing (scRNA-seq) of mouse ovaries from reproductive young (Y, 4.5-mo, n = 5), peri-estropause (M, 10.5-mo, n = 7), and post-estropause (O, 15.5-mo, n = 5) stage (Fig. 1*A*). The estrous cycle of each mouse was assessed daily for a minimum of 3 wk to precisely define their reproductive status (15). The reproductive young mice, exhibiting regular cycles every 4 to 5 d for at least 2 wk, and the post-estropausal mice reaching acyclicity (no cycle within 9 d), were selected for ovarian scRNA-seq (*SI Appendix,* Fig. S1*A*). Among the peri-estropausal mice, 3 out of 7 displayed regular cycles, while the remaining four showed irregular cycles (two contiguous cycles of 5 to 8 d, *SI Appendix*, Fig. S1*A*). All ovary samples were collected when the mice were in diestrus.

We obtained 53,119 single-cell transcriptomes that passed quality control for downstream analysis and applied uniform manifold approximation and projection (UMAP) analysis to resolve the cell type distribution (Fig. 1*B* and *SI Appendix*, Fig. S1*B*). Eleven distinct cell clusters were identified through unsupervised clustering. Based on well-defined cell type–specific markers, the major cell categories of the ovary were annotated, including oocytes (O, *Ooep*^+^), 3 groups of granulosa cells (GC), luteal cells (LC, *Sfrp4*^+^), theca cells (TC, *Aldh1a1*^+^), stromal cells (SC, *Dcn*^+^), epithelial cells (EPC, *Epcam*^+^), endothelial cells (EC, *Cdh5*^+^), and 2 groups of immune cells (IC) (Fig. 1 *B* and *C*). The 3 GC clusters, *Amh*-high (GC-A), *Inhbb*-high (GC-I), and *Nppc*-high (GC-N), differed by expression of hormone-related genes, suggesting that they may represent granulosa cells at different follicular development stages (*SI Appendix*, Fig. S1*C*) (16). LC expressed markers of both theca (*Aldh1a1*, *Star*) and granulosa cells (*Nr5a2*) (17), suggesting that these luteal cells are derived from either granulosa or theca cells undergoing luteinization (Fig. 1*C*). Immune clusters included NK/T cells (*Cd3g*^+^, *Nkg7*^+^) and macrophage/B cells (*Lyz2*^+^, *Cd74*^+^).

### Altered Cell Type Composition during Mouse Ovarian Aging.

To examine age-related shifts in ovarian cell composition, we compared the proportions of major cell types across reproductive young (Y), peri-estropause (M), and post-estropause (O) stages and observed age-dependent changes in several cell types (Fig. 1*D*). Oocytes decreased sharply from 6.7% in the young stage to 0.1% in post-estropausal ovaries, representing a near-complete depletion of the germ cell population. Similarly, *Amh*-high granulosa cells and theca cells exhibited substantial declines of 5.6-fold and 1.57-fold, respectively, which could be attributed to the decline of follicle numbers during aging. Stromal and endothelial cells remained stable until peri-estropause but dropped significantly in post-estropause ovaries. In contrast, epithelial and immune cell populations increased with age, aligning with reported epithelial thickening and immune infiltration in aged ovaries (18).

### Global Transcriptional Change during Mouse Ovarian Aging.

Increased transcriptional noise is a signature of aging (19, 20). To evaluate this signature during ovarian aging, we calculated the distribution of the coefficient of variation (CV) of highly variable genes within each cell cluster across different age groups. Our analysis revealed a significant increase in transcriptional noise in peri-estropause (M) and post-estropause (O) ovaries compared to the young ovaries (Y) (*SI Appendix*, Fig. S1 *D* and *E*). Moreover, we performed the age-relevant CV analysis to examine the aging-associated transcriptional variability in different cell types. Granulosa and theca cells showed higher CVs than other somatic cell types (*SI Appendix*, Fig. S1 *F* and *G*), suggesting a higher vulnerability of follicular cells to ovarian aging.

To characterize transcriptional changes across ovarian aging, we performed pairwise differentially expressed gene (DEG) analyses between reproductive young mice and mice of other ages. A total of 783 DEGs were identified when comparing peri-estropause to young ovaries (M vs. Y), with 317 upregulated and 466 downregulated (*SI Appendix*, Fig. S1*H*). The extent of transcriptional divergence increased substantially in mice approaching post-estropause (O vs. Y), with 3,668 DEGs identified (1,394 upregulated and 2,274 downregulated), representing a 4.7-fold increase compared to the M vs. Y comparison. These results indicate a progressive and widespread transcriptional downregulation during advanced aging (*SI Appendix*, Fig. S1*H*). Notably, 60% (192/317) of the upregulated DEGs and 77% (359/466) of the downregulated DEGs in peri-estropause (M) ovaries remained altered in the same direction in post-estropause (O) ovaries compared to young ovaries. This finding indicates that most age-related gene expression changes observed in peri-estropause persist through post-estropause, suggesting their sustained contribution to the functional decay of the ovary. Cell type–specific DEG analysis further showed that there were more DEGs in luteal cells (LC), endothelial cells (EC), and oocytes at the peri-estropause stage, and in *Amh*-high granulosa cells (GC-A), LC, and stromal cells (SC) at the post-estropause stage (*SI Appendix*, Fig. S1*I*).

Interestingly, numerous aging-associated DEGs, especially in the comparison between O vs. Y, were shared across cell types (*SI Appendix*, Fig. S2 *A* and *B*), similar to what we observed during ovarian aging in humans (21). To further investigate the extent of coordinated changes in transcriptomes during ovarian aging, we calculated pairwise cosine similarity of aging-associated DEGs in the ovary and compared it to those from other tissues using the Tabula Muris Senis dataset, including the heart, lung, liver, kidney, muscle, spleen, marrow, and brain. Ovarian cells in the post-estropause stage (O) exhibited significantly higher transcriptomic coordination compared to those in the peri-estropause stage (M) (Fig. 1*E*). Notably, compared with other tissues, ovarian cells (O vs. Y) exhibited significantly higher coordination, despite post-estropause ovaries (15.5 mo) being chronologically younger than the other tissues analyzed (18 to 30 mo) (Fig. 1*E* and *SI Appendix*, Fig. S2 *C*–*K*). These results indicate that a high level of coordination is a distinctive characteristic of ovarian aging in both mice and humans (21).

### Monotonic Transcriptomic Signatures of Mouse Ovarian Aging across Reproductive Lifespan.

To further characterize transcriptomic dynamics during ovarian aging, we identified DEGs of across age groups within each cell type (MAST test, min.pct = 0.25, |log_2_ FC| > 0.25, adjusted *P* < 0.05). Based on LOESS-regressed age-associated trends, these DEGs were categorized into four trajectory patterns: monotonic up (MU), monotonic down (MD), up–down (UD), and down–up (DU) (*SI Appendix*, Fig. S3*A*). We then focused on DEGs that monotonically increased or decreased with age, termed monotonic DEGs (MDEGs). Oocytes were excluded due to the limited number (eight) in post-estropause samples. This analysis identified 773 upregulated and 1,679 downregulated MDEGs across 10 ovarian somatic cell types. Consistent with the high transcriptomic coordination observed in pairwise DEG analysis (Fig. 1*E* and *SI Appendix*, Fig. S2), numerous MDEGs were shared across cell types, with 56 upregulated and 189 downregulated MDEGs common to at least four cell types (*SI Appendix*, Fig. S3 *B*–*D*). To identify the most significantly altered MDEGs in each cell type, we calculated the log_2_ fold change (O vs. Y) of MDEGs and identified the top five most upregulated and downregulated genes within each cell type (*SI Appendix*, Fig. S3*E*). Several genes from the activator protein 1 (AP-1) superfamily of transcription factors, including *Fos*, *Fosb*, *Atf3*, and *Junb*, were consistently among the most downregulated MDEGs across various cell types. Notably, in primate ovarian aging, *FOSB* was identified as a hub gene regulating the downregulated gene subset in granulosa cells (20). Additionally, *Fos* null mice fail to ovulate or form a corpus luteum (CL) even with exogenous gonadotropin stimulation (22). The downregulation of *Fosb* and *Fos* across cell types during ovarian aging suggests that these genes may serve as key mediators in regulating ovarian function.

Gene Ontology (GO) analysis of all MDEGs revealed enriched pathways shared across different cell types, including oxidative phosphorylation, responses to oxidative stress and hormones, regulation of protein stability and ribosome-related processes (Fig. 1*F*). To investigate the impact of ovarian aging on these pathways, we examined the pathway activity scores based on gene set variation analysis (GSVA) or corresponding gene lists (*SI Appendix*, Table S1). The results showed a continuous increase in the steroid hormone biosynthesis score, and continuous decreases in the ribosome-related signaling and the unfolded protein response scores during ovarian aging across almost all cell types (Fig. 1*G* and *SI Appendix*, Figs. S3 *F* and *G* and S4 *A*–*C*). Notably, oxidative phosphorylation (OXPHOS) scores declined with age overall (Fig. 1*H*) and across all cell types but GC-I, GC-N, and luteal cells (LC) (*SI Appendix*, Fig. S4*D*). Dysfunction of mitochondrial OXPHOS has been a hallmark signature of aging in various organs (23). Interestingly, almost all mitochondrial-encoded OXPHOS genes decreased, while gene set score of nuclear-encoded OXPHOS genes increased across most cell types (*SI Appendix*, Fig. S4 *E*–*H*). Thus, the apparent increase in OXPHOS pathway scores in GC and LC reflects compensatory upregulation of nuclear-encoded genes in the context of declining mitochondrial function, rather than enhanced mitochondrial activity. The imbalance in the expression between mitochondria- and nuclear-encoded OXPHOS subunits is predicted to cause stoichiometry disruption of OXPHOS machinery, leading to OXPHOS dysfunction. Indeed, we found a significant decline in mitochondrial membrane potential in aged ovarian cells (Fig. 1*I*). These results suggest that the transcriptional dysregulation underlies disrupted mitochondrial homeostasis and may reflect defects in nuclear-mitochondrial communication during mouse ovarian aging.

### Single-Cell Transcriptomic Profiling of Ovary-Specific Senescent Cells.

**Fig. 2.**
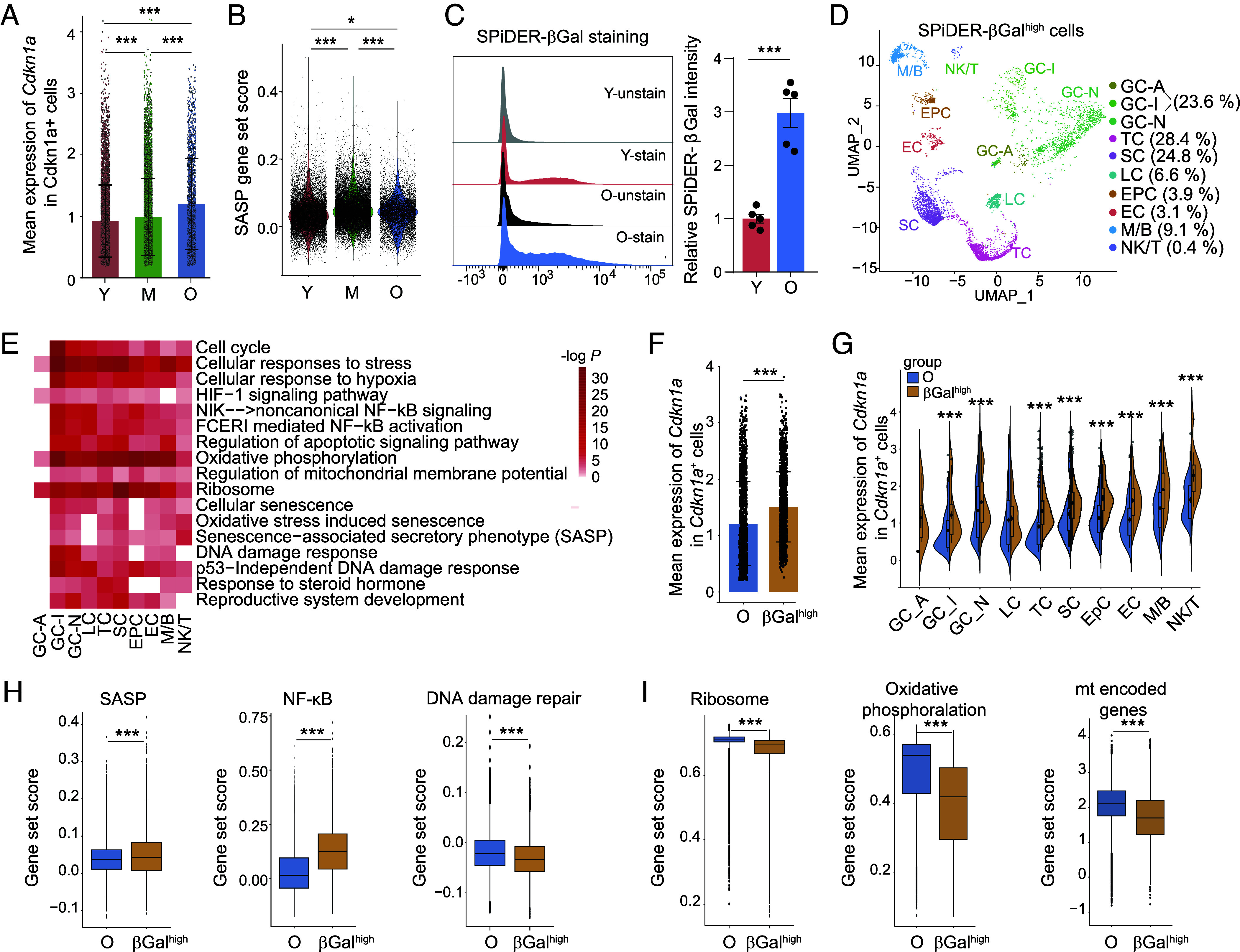
Senescence cell accumulation during ovarian aging and scRNA-seq profiling of ovary-specific senescent cells. (*A*) Bar plots showing the expression of *Cdkn1a* for the cells that express the gene at each age group (Mean ± SEM, Wilcoxon test, ***Padj < 0.001). (*B*) Violin plots showing the SASP gene set score at each age group (Wilcoxon test, *Padj < 0.05, ***Padj < 0.001). (*C*) Flow cytometry analysis of SPiDER-βGal-stained mouse ovarian cells. The *Left* panel showed the representative histograms of the SPiDER-βGal signal captured in the GFP channel. The *Right* panel showed the statistical comparison of SPiDER-βGal intensity between young (Y) and post-estropause (O) ovaries. SPiDER-βGal intensity for each sample was calculated by subtracting the mean GFP intensity in the unstained sample from the mean GFP intensity in the stained sample. Background-removed intensities were normalized to the young group. (Mean ± SEM; *t* test; ***P < 0.001; n = 5). (*D*) UMAP plots showing the cell types of βGal^high^ cells in post-estropause mouse ovary. (*E*) Heatmap showing representative GO terms of DEGs (MAST test) between βGal^high^ cells and aged-matched ovarian cells in each cell type. (*F*) Bar plots showing the expression of *Cdkn1a* for the cells that express the gene in βGal^high^ cells and aged-matched ovarian cells (O). (Mean ± SEM, Wilcoxon test, ***Padj < 0.001). (*G*) Violin plots showing expression of *Cdkn1a* for the cells that express the gene in βGal^high^ cells and aged-matched ovarian cells (O) across cell types. (Wilcoxon test, ***Padj < 0.001). (*H*) Box plots showing the gene set score of senescence-associated secretory phenotype (SASP), NK-κB pathway, and DNA damage repair in βGal^high^ cells and aged-matched ovarian cells (O) (Wilcoxon test, ***Padj < 0.001). (*I*) Box plots showing the gene set score of ribosome, oxidative phosphorylation, and mitochondrial (mt) encoded genes in βGal^high^ cells and aged-matched ovarian cells (O) (Wilcoxon test, ***Padj < 0.001).

Cellular senescence is a hallmark of tissue aging (24). To assess senescent cell burden during ovarian aging, we examined the expression of classical senescence-associated cell cycle arrest markers, *Cdkn2a* (p16) and *Cdkn1a* (p21). Similar to human ovarian cells (21), only a small fraction of mouse ovarian cells expressed *Cdkn2a* (average 1.8%), while a larger proportion expressed *Cdkn1a* (average 25.2%). *Cdkn1a* expression significantly increased with age within *Cdkn1a*^+^ ovarian cells (Fig. 2*A*), with the strongest increases in *Inhbb*-high (GC-I, 1.2-fold) and *Nppc*-high (GC-N, 1.8-fold) granulosa cells in post-estropause (O) ovaries compared with young (Y) ones (*SI Appendix*, Fig. S5*A*). We next assessed senescence-associated secretory phenotype (SASP) gene set scores using published gene lists (25, 26), and found significant increases in all ovarian cells from peri- (M) and post-estropause (O) ovaries compared to young ones (Y), with peri-estropause ovaries showing the highest score (Fig. 2*B*). Among cell types, GC-I and GC-N exhibited a consistent increase in SASP scores throughout the aging process (*SI Appendix*, Fig. S5*B*). In line with these transcriptomic changes, we observed significantly elevated senescence-associated β-galactosidase (SA-β-gal) activity (2.98-fold higher), a canonical marker of cellular senescence, in live ovarian cells isolated from aged mice compared to cells from young mice, as measured by SPiDER-βGal staining (Fig. 2*C*). The increased abundance of SPiDER-βGal^high^ cells in aged ovaries likely reflects the accumulation of age-associated senescent cells, superimposed on a smaller baseline population of transiently senescent cell.

To further characterize ovary-specific senescent cells, we isolated live SPiDER-βGal^high^ cells from post-estropausal ovaries (O) and performed single-cell RNA sequencing. SPiDER-βGal^high^ cells (27) are interpreted as enriched for senescent-like states rather than representing a strictly defined senescent population. We mapped a total of 5,805 the SPiDER-βGal^high^ cells to our original mouse ovary single-cell sequencing dataset and found that granulosa (GC, 23.6%), theca (TC, 28.4%), and stromal cells (SC,24.8%) were predominant among ovarian senescent cells (Fig. 2*D*). We then identified ovarian senescence-associated DEGs (O-SenDEGs) by comparing SPiDER-βGal^high^ cells to age-matched ovarian cells (O) across cell types (*SI Appendix*, Fig. S5*C*). A substantial number of O-SenDEGs were shared across cell types, with 209 genes upregulated and 214 genes downregulated in at least five ovarian cell types (*SI Appendix*, Fig. S5*D*). GO analysis revealed that the O-SenDEGs were enriched in hallmarks of cellular senescence including cell cycle regulation, cellular response to hypoxia (HIF-1 signaling pathway), NF-κB signaling pathway, SASP, and DNA damage response (Fig. 2*E*). SPiDER-βGal^high^ cells exhibited significantly higher expression of cell cycle arrest-associated markers including *Cdkn1a* (p21), *Cdkn2a* (p16), *Cdkn2b* (p15), and *Cdkn1b* (p27), compared to age-matched ovarian cells (O) (Fig. 2*F* and *SI Appendix*, Fig. S5*E*). However, even among SPiDER-βGal^high^ cells, only a small fraction expressed *Cdkn2a* (1.96%) and *Cdkn2b* (2.84%), while larger proportions expressed *Cdkn1a* (27.68%) and *Cdkn1b* (31.49%), highlighting the current challenges in defining specific markers exclusive to senescent cells in vivo (14). Notably, *Cdkn1a* showed consistently elevated expression in SPiDER-βGal^high^ cells compared to controls across all cell types (Fig. 2*G*), along with a progressive increase with aging (Fig. 2*A*), suggesting it is a more reliable cell cycle arrest-related senescence marker in the mouse ovary. The proportion of SPiDER-βGal^high^ cells expressing *Cdkn1a* varied across cell types, with immune cells showing the highest overlap (*SI Appendix*, Fig. S5*F*), reflecting heterogeneity in senescence markers across cell types. In addition, SPiDER-βGal^high^ cells exhibited increased SASP gene set score and higher NF-κB transcriptional activity, along with reduced DNA damage repair score (Fig. 2*H* and *SI Appendix*, Fig. S5 *G*–*I*). These findings suggest that the SPiDER-βGal^high^ cells with age-related increase in βGal activity can represent senescent cells in the mouse ovary. Additionally, SPiDER-βGal^high^ cells exhibited downregulation of ribosomal and oxidative phosphorylation pathways across all cell types (Fig. 2*I* and *SI Appendix*, Fig. S5 *J* and *K*), with multiple mitochondrial-encoded genes (*mt-Nd2, mt-Co1, mt-Atp8, mt-Nd3, mt-Co2, mt-Nd1,* and *mt-Co3*) among the most consistently and strongly reduced (Fig. 2*I* and *SI Appendix*, Fig. S5 *C*, *D*, and *L*). These results align with established transcriptomic patterns of ovarian aging (Fig. 1 *G* and *H*), suggesting that mitochondrial and ribosomal dysfunction are closely associated with ovary-specific cellular senescence; however, their precise causal role remains to be determined.

### Altered Cellular Communications during Ovarian Aging.

**Fig. 3.**
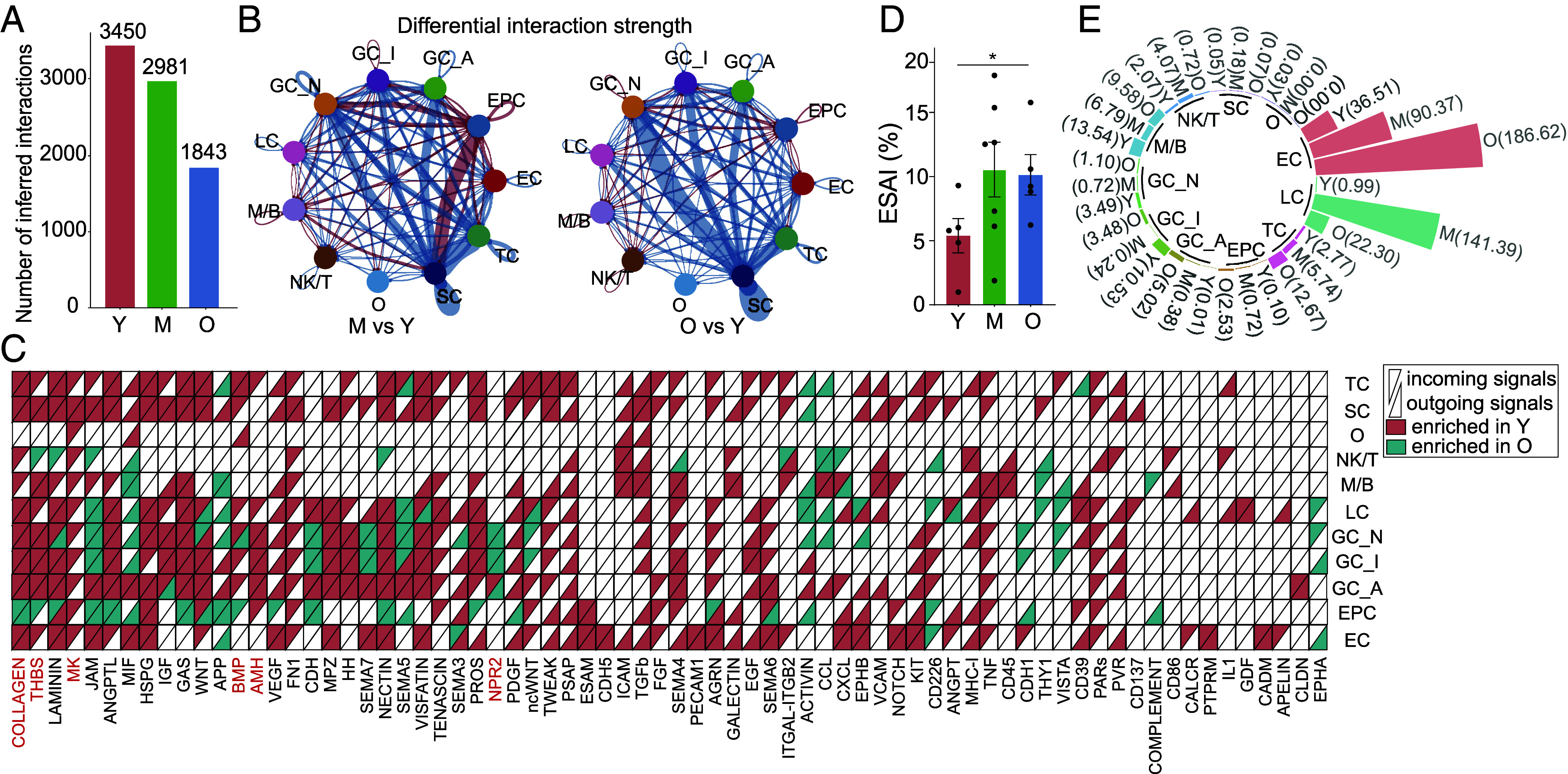
Reduction in intercellular interactions and increase in extracellular vesicle secretion among ovarian cells during aging. (*A*) Bar plots showing the number of intercellular interactions among ovarian cells at each age group. (*B*) Circle plots showing the change of interaction strength in comparison between peri-estropause vs. young ovaries (M vs. Y, *Left*) and between post-estropause vs. young ovaries (O vs. Y, *Right*). Blue lines indicate that the displayed communication is decreased in peri-estropause (*Left*) or post-estropause ovaries (*Right*), while red lines indicate that communication is increased in peri-estropause (*Left*) or post-estropause ovaries (*Right*). The arrows indicate the direction of intercellular communication. (*C*) Heat map showing the outgoing and incoming signaling pathways significantly enriched in young (Y) or post-estropause (O) ovaries for each cell type. (*D*) sEV secretion activity index (ESAI) of each ovary sample from different groups. (Mean ± SEM; *t* test; *P < 0.05). (*E*) ESAI of different ovarian cell types in young (Y), peri-estropause (M), and post-estropause (O) ovaries.

Aging is associated with progressive changes in intercellular communication (24). Using CellChat (28), we found that both the total number and overall strength of interactions among ovarian cell types declined with age (Fig. 3 *A* and *B*). Specifically, communication among *Nppc*-high granulosa (GC-N), stromal (SC), and theca (TC) cells declined sharply during aging, while epithelial cell interactions increased (Fig. 3*B*).

To elucidate altered pathways of cell–cell communication during mouse ovarian aging, we conducted a comparative analysis of communication probabilities among all pairs of cell groups in post-estropause (O) and peri-estropause (M) ovaries compared to young ovaries. We identified 73 pathways in peri-estropausal (M) ovaries and 72 in post-estropausal ovaries (O) that significantly differed in communication probability from those in young ovaries (Fig. 3*C* and *SI Appendix*, Fig. S6*A*). Notably, pathways critical for follicle development, such as BMP signaling (29), peaked in young ovaries, decreased in peri-estropause (M) ovaries, and became depleted in post-estropause (O) ovaries (*SI Appendix*, Fig. S6 *B* and *C*). AMH signaling from granulosa cells (GC) also steadily declined with age, aligning with the concurrent reduction in growing follicles during ovarian aging (*SI Appendix*, Fig. S6*D*). Interestingly, NPR2 signaling, crucial for maintaining oocyte meiotic arrest (30), was found to be more abundant in aged ovaries (*SI Appendix*, Fig. S6*E*). Analysis of ligand (*Nppc*) and receptor (*Npr2*) expression across age groups revealed an increase in *Nppc* and a decrease in *Npr2* with aging (*SI Appendix*, Fig. S6*E*). This dysregulation of NPR2 signaling may contribute to abnormalities in follicular development, potentially leading to impaired oocyte meiosis and increased aneuploidy observed in reproductive aging. Additionally, we found the midkine (MK) signaling, involved in follicle maturation (31), was primarily derived from SC and significantly declined with aging (*SI Appendix*, Fig. S6*F*). These results indicate that the gradually diminishing interactions among oocytes, GC, and SC during ovarian aging may contribute to the dysfunction of folliculogenesis during aging. Moreover, collagen signaling and thrombospondin (THBS) signaling, the core components of extracellular matrix (ECM) biology (32), showed significantly higher communication probability in most cell types in the young ovary, indicating ECM disruption during aging (Fig. 3*C* and *SI Appendix*, Fig. S6*G*). In contrast, epithelial cells exhibited a significantly higher communication probability of these signaling in the aged ovary, which may explain the reported outgrowth of ovarian surface epithelium in aging ovaries (18). Furthermore, cellular communication through signaling pathways related to inflammation and immune regulation (33–37), such as COMPLEMENT, VISTA, CCL, CD226, and THY1, were notably increased in aged immune cells (Fig. 3*C*), reflecting inflammaging and compromised immune homeostasis in old ovaries.

In addition to the intercellular communication mediated by direct ligand–receptor interaction, we evaluated indirect communication through small extracellular vesicles (sEV) in the mouse ovary using SEVtras (38). The sEV secretion activity index (ESAI) significantly increased with reproductive aging (Fig. 3*D*), consistent with the current knowledge that aging is accompanied by increased EV release, attributing to elevated SASP, compromised immune response, inflammation, and tissue damage (39). Among cell types, endothelial cells (EC), luteal cells (LC), immune cells (IC), and theca cells (TC) showed higher sEV secretion activity, with EC contributing most to the age-related increase (Fig. 3*E*). While the function of EC-derived sEVs is not fully understood, given that EC serves as the interface between circulating blood and the vascular wall, they may communicate with cells from both sides, potentially impacting vascular integrity and inflammation.

### Hormonal Dysregulation in Granulosa Cell Subpopulations during Ovarian Aging.

**Fig. 4.**
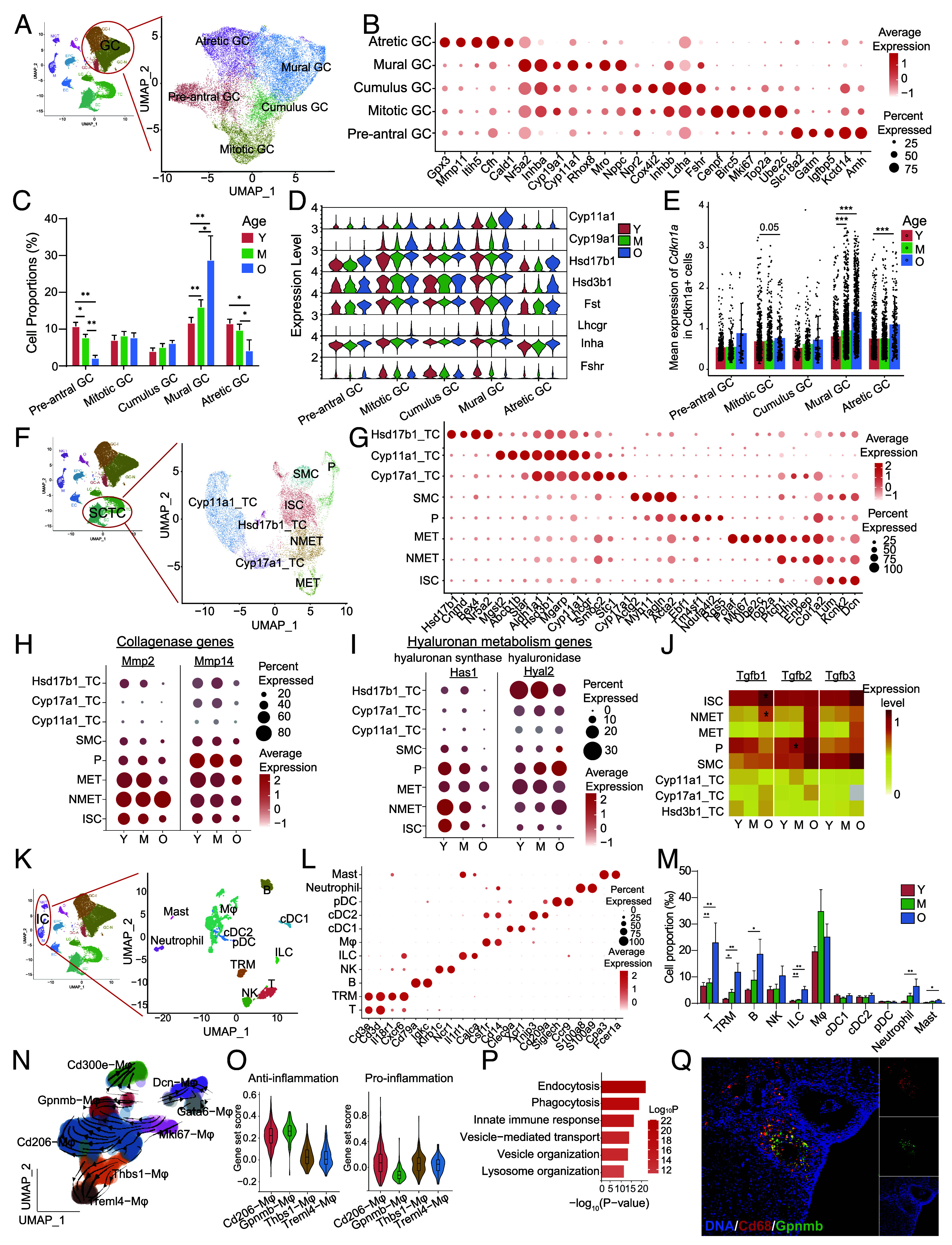
Alterations in granulosa, stromal/theca, and immune cell subpopulations during ovarian aging. (*A*) UMAP plots showing the subpopulations of mouse granulosa cells (GC). (*B*) Dot plots showing the expression of representative genes for each subpopulation of GC. (*C*) Bar plots showing the proportion of each GC subpopulation in young (Y), peri-estropause (M), and post-estropause (O) ovaries. (Mean ± SEM; Permutation test; *Padj < 0.05, **Padj < 0.01). (*D*) Violin plots showing the expression of hormone-related genes in mouse granulosa cells at each age group. (*E*) Bar plots showing the expression of *Cdkn1a* in each subpopulation of GC for the cells that express the gene at each age group. (Mean ± SEM, Wilcoxon test, ***Padj < 0.001). (*F*) UMAP plots showing the subpopulations of mouse stromal and theca cells (SCTC). ISC, interstitial stromal cells; SMC, smooth muscle cells; P, pericytes; MET, mitotic early theca cells; NMET, nonmitotic early theca cells. Cyp17a1-TC, *Cyp17a1*-high theca cells; Cyp11a1-TC, *Cyp11a1*-high theca cells; Hsd17b1-TC, *Hsd17b1*-high theca cells. (*G*) Dot plots showing the expression of representative genes for each subcluster of SCTC. (*H*) Dot plots showing the expression of collagenase genes in subclusters of SCTC. (*I*) Dot plots showing the expression of hyaluronan metabolism genes in subclusters of SCTC. (*J*) Heatmap showing the expression of *Tgfb* genes in each subclusters of SCTC at each age group. (Wilcoxon test, *Padj < 0.05 when comparing with young ovary). (*K*) UMAP plots showing the subpopulations of mouse immune cells. B, B cells; T, T cells; NK, natural killer cells; ILC, innate lymphoid cells; Mφ, macrophages; cDC1, type 1 conventional dendritic cells; cDC2, type 2 conventional dendritic cell; pDC, plasmacytoid dendritic cell; TRM, tissue-resident memory T cells. (*L*) Dot plots showing the expression of representative genes for each subcluster of immune cells. (*M*) Bar plots showing the proportion of each immune cell type in young (Y), peri-estropause (M), and post-estropause (O) ovaries. (Mean ± SEM; Permutation test; *Padj < 0.05, **P < 0.01). (*N*) RNA velocity stream for macrophages projected onto the UMAP embedding. (*O*) Violin plots showing M2 anti-inflammatory (*Left*) and M1 pro-inflammatory (*Right*) gene set score in different macrophages. (*P*) GO analysis showing the functions that maker genes of Gpnmb-Mφ were enriched in. (*Q*) Representative image showing immunofluorescence staining of *Gpnmb*-positive macrophages in young ovaries.

Granulosa cells (GC) were the largest cell cluster in the mouse ovary (Fig. 1*B*) and showed the greatest transcriptional variability associated with aging (*SI Appendix*, Fig. S1 *F* and *G*). Given their high cellular heterogeneity, we further subclustered them to analyze transcriptional changes across different GC subtypes throughout the reproductive lifespan. Unsupervised clustering divided GC into five main subpopulations: pre-antral GC (*Amh*^+^, *Gata*^+^), atretic GC (*Itih5*^+^, *Cald1*^+^), mitotic GC (*Top2a*^+^, *Mki67*^+^), cumulus GC (*Ldha*^high^, *Cox4i2*^+^), mural GC (*Nppc*^high^, *Mro*^+^) (Fig. 4 *A* and *B*) (40–42). During aging, the proportions of pre-antral GC and atretic GC decreased significantly, while mural GC increased significantly during aging (Fig. 4*C*). Interestingly, this increase was largely attributed to the increase of *Lhcgr*^+^ mural GC in post-estropause ovaries (*SI Appendix*, Fig. S7 *A* and *B*). Consistently, *Lhcgr* expression significantly increased in mural GC during ovarian aging (3.4-fold increase in O vs. Y), ranking among the top three upregulated DEGs alongside *Rhox8* and *Cyp11a1* (*SI Appendix*, Fig. S7 *C*–*E*). Typically, mural GC express high levels of *Lhcgr*, encoding the receptor for both luteinizing hormone and choriogonadotropin, exclusively in preovulatory follicles (43). Elevated LH receptor expression was also observed in early-stage GC in PCOS ovaries, leading to premature luteinization of GC during follicular development (44). Further investigation is needed to determine whether the mural GC expressing a high level of *Lhcgr* may contribute to the age-related decline in mouse fertility.

To unravel the transcriptome changes underlying the age-related GC subpopulation dynamics, we identified the MDEGs within each subcluster (*SI Appendix*, Fig. S7*F*). Notably, *Adamts1*, crucial for ovulation, consistently decreased across all GC subpopulations. Impaired ovulation due to mature oocytes remaining trapped in ovarian follicles has been reported in *Adamts1*-null mice (45). Additionally, we found increased expression of *Fst,* which encodes follistatin, a glycoprotein that binds activin to suppress FSH secretion and stimulates luteinization (46). GO analysis of all MDEG in GC during ovarian aging revealed enrichment in common pathways shared across GC subpopulations, including oxidative phosphorylation, cellular response to stress, vasculature development, and response to hormones (*SI Appendix*, Fig. S7*G*). As key producers of ovarian steroid hormones such as estradiol and progesterone, GC, in cooperation with theca cells (TC), regulate the production of FSH and LH from the pituitary gland through both negative and positive feedback mechanisms. During aging, we observed common changes in the expression of hormone synthesis-related genes across GC subpopulations, including a decrease in *Fshr* and increases in *Cyp11a1*, *Hsd3b1*, *Cyp19a1*, *Hsd17b1*, and *Inha* (Fig. 4*D* and *SI Appendix*, Fig. S7*H*). Notably, post-estropausal mice generally maintain moderate to high circulating levels of 17β-estradiol and progesterone, and relatively lower levels of LH and FSH, which is different to the distinctive hormonal levels in post-menopausal women, i.e., very low levels of 17β-estradiol and progesterone, and significantly elevated levels of FSH and LH (4). These results suggest that differences in hormone-related gene expression changes may underlie the dissimilarity between mice and humans during ovarian aging (Fig. 4*D* and *SI Appendix*, Fig. S7*I*).

Of note, we observed concordant increases in *Cdkn1a* expression across all GC subpopulations, with significant changes in mitotic, mural, and atretic GC, especially in post-estropause (O) ovaries (Fig. 4*E*). Furthermore, all GC subpopulations, except for pre-antral GC, showed a significant increase in SASP score (*SI Appendix*, Fig. S7*J*). These findings suggest that senescence burden elevates across all GC subtypes throughout reproductive lifespan, which may in turn contribute to ovarian aging.

### Extracellular Matrix Remodeling and Fibrogenesis in Stromal/Theca Subpopulations during Ovarian Aging.

Ovarian stromal cells (SC) are a heterogeneous population that provide structural support for folliculogenesis and engage in bidirectional paracrine signaling with follicles (47), while theca cells (TC) are thought to be recruited from surrounding stromal tissue during follicle development (48). To characterize their heterogeneity and aging-related changes, we classified mouse SC and TC into eight subpopulations by unsupervised clustering, including interstitial stromal cells (ISC, *Dcn*^+^, *Lum*^+^, *Kcnk2*^+^), smooth muscle cells (SMC, *Acta*^+^, *Actg*^+^), pericytes (P, *Rgs5*^+^, *Ebf1*^+^), two groups of early TC (*Enpep*^+^, *Hhip*^+^), and three groups of steroidogenic TC (*Star*^+^, *Aldh1a1*^+^) (Fig. 4 *F* and *G*). Mitotic early theca cells (MET) and nonmitotic early theca cells (NMET) shared most of the marker genes, except for proliferation-related genes such as *Top2a*, *Mki67*, and *Ube2c*, which were exclusively expressed in MET. Steroidogenic TC were subdivided into *Cyp17a1*-high (Cyp17a1-TC), *Cyp11a1*-high (Cyp11a1-TC), and *Hsd17b1*-high theca cells (Hsd17b1-TC), each expressing high level of key enzymes involved in androgen biosynthesis in TC. Early TC were thought to form the theca interna of pre-antral follicles while steroidogenic TC was responsible for the theca interna of antral follicles (49). Notably, ovarian aging was marked by a pronounced decrease in early TC (from 24% to 4%; *SI Appendix*, Fig. S8*A*), consistent with the loss of growing follicles post-estropause. These results indicate that both SC and TC comprise highly differentiated subtypes with distinct structural and steroidogenic roles in the ovary.

Increased stiffness in the aging ovary is associated with changes in stromal ECM components including collagen and hyaluronan (50). Excessive collagen accumulation was reported to be the primary cause of increased stiffness in the ovary. We found SC were the major cell type in the mouse ovary that robustly expressed collagen-associated genes, with Types I, II, IV, V, and VI being the most abundant (*SI Appendix*, Fig. S8 *B* and *C*). Further analysis of stromal subtypes showed that these collagen genes were modestly expressed in steroidogenic TC, but highly expressed in ISC, early TC, and pericytes (*SI Appendix*, Fig. S8*D*). Collagen gene expressions in ISC unexpectedly decreased while they were well maintained in early TC and pericytes during ovarian aging. We then evaluated collagenase expression and found *Mmp2* was uniquely expressed in SC, while *Mmp14* was more broadly expressed (*SI Appendix*, Fig. S8*E*). Both *Mmp2* and *Mmp14* levels diminished in aged ISC and MET, suggesting impaired collagen degradation may lead to collagen accumulation in post-estropause (O) ovaries (Fig. 4*H*). Since hyaluronic acid (HA) reduction is another mechanism causing increased stiffness, we next focused on HA metabolism genes including three hyaluronan synthases (*Has1*, *Has2*, and *Has3*) and four hyaluronidases (*Hyal1*, *Hyal2*, *Cemip*, and *Cemip2*). SC showed a relatively high expression of *Has1* comparing to other ovarian cell types (*SI Appendix*, Fig. S8*F*). Interestingly, we found hyaluronidase gene *Cemip* was predominantly expressed in luteal cells (LC), which may explain the minimal presence of HA in the corpus luteum (50). During aging, *Has1* expression declined in ISC, pericytes, and NMET, while *Hyal2* increased in pericytes and NMET, indicating dysregulated hyaluronan synthesis and degradation in the post-estropause (O) ovary (Fig. 4*I*). Furthermore, we observed a significant increase in the expression of *Tgfb1*, a central mediator of collagen deposition, hyaluronan synthesis, and fibrogenesis, in ISC and NMET in the old ovary, suggesting their contribution to fibrogenesis during mouse ovarian aging (Fig. 4*J* and *SI Appendix*, Fig. S8 *G*–*I*).

### Functional Decline in Phagocytosis-Associated Macrophages during Ovarian Aging.

To better resolve immune cell heterogeneity in mouse ovaries, we performed unsupervised clustering of the initial IC clusters and identified diverse lymphoid cells, including B cells (B, *Cd79a*^+^), T cells (T, *Cd3*^+^), natural killer cells (NK, *Klrb1c*^+^), and innate lymphoid cells (ILC, *Il1rl1*^+^), as well as myeloid populations, such as macrophages (Mφ, *Cd14*^+^), type 1 conventional dendritic cells (cDC1, *Xcr1*^+^, *Clec9a*^+^), type 2 conventional dendritic cell (cDC2, *Tnip3*^+^, *Cd209a*^+^), plasmacytoid dendritic cells (pDC, *Siglech*^+^), neutrophils (*S100a8*^+^, *S100a9*^+^), and mast cells (*Cpa3*^+^) (Fig. 4 *K* and *L*).

During reproductive aging, we observed a progressive accumulation of various immune cell populations across the young (Y), peri-estropause (M), and post-estropause (O) ovaries. Specifically, when comparing the post-estropause to the young ovaries, the proportions of neutrophils (7.9-fold), ILCs (5-fold), mast cells (3.1-fold), T cells (3-fold), B cells (3-fold), and NK cells (1.7-fold) were significantly higher, whereas the total macrophage proportion remained unchanged (Fig. 4*M*). Eight macrophage subpopulations were further identified through unsupervised cluster analysis, namely Cd206*-*Mφ (*Mrc1*^+^, *Pf4*^+^), Gpnmb-Mφ (*Atp6v0d2*^+^, *Gpnmb*^+^), Cd300e-Mφ (*Cd300e*^+^, *Asb2*^+^, *Fcrl5*^+^), Thbs1-Mφ (*Thbs1*^+^, *Ccr2*^+^), Treml4-Mφ (*Treml4*^+^, *Ace*^+^), Mki67-Mφ (*Mki67*^+^, *Top2a*^+^), Dcn-Mφ (*Dcn*^+^, *Mgp*^+^), Gata6-Mφ (*Gata6*^+^, *Esr2*^+^) (*SI Appendix*, Fig. S9 *A* and *B*). Utilizing RNA velocity analysis (51), we inferred cellular state transitions among these subpopulations. This analysis revealed a clear directional flow from proliferative Mki67-Mφ to Cd206-Mφ and then diverged into either Gpnmb-Mφ or Thbs1- and Treml4-Mφ (Fig. 4*N*). Using signature genes of “Pro-inflammatory” M1 and “Anti-inflammatory” M2 macrophages (52), we found the coexpression of both M1 and M2 gene signatures in Cd206-Mφ and Cd300e-Mφ (Fig. 4*O*), consistent with previous studies (53), suggesting a more complex phenotype of in vivo macrophage compared to in vitro activation state of macrophages. Notably, Treml4-Mφ and Thbs1-Mφ exhibited strong pro-inflammatory signatures, while Gpnmb-Mφ exhibited strong anti-inflammatory signatures (Fig. 4*O*). We also noticed the proportion of pro-inflammatory Treml4- and Thbs1-Mφ increased with reproductive aging, while the proportion of anti-inflammatory Gpnmb-Mφ decreased (*SI Appendix*, Fig. S9*C*). To understand the potential function of Gpnmb-Mφ, we compared its gene expression with that of other macrophages and found that genes highly expressed in Gpnmb-Mφ were enriched in functions including phagocytosis (Fig. 4*P*), suggesting Gpnmb-Mφ is a type of macrophage with phagocytotic capacity. Consistently, the expression of genes involved in phagocytosis including *Mertk* and *Trem2* was stronger in Gpnmb-Mφ compared to other macrophages, while the pro-inflammatory gene *Il1b* was almost absent (*SI Appendix*, Fig. S9 *D* and *E*). During ovarian aging, we saw a trend of declined expression of *Mertk* and *Trem2* in Gpnmb-Mφ (*SI Appendix*, Fig. S9*F*), suggesting a loss of phagocytotic capacity in this macrophage. We further verified the presence of this Mφ in the corpus luteum, as well as in potential atretic follicles or cysts, where apoptotic or defective cells were abundant (Fig. 4*Q* and *SI Appendix*, Fig. S9*G*). Taken together, we identified a unique macrophage in the mouse ovary that declined in cell proportion and phagocytosis capacity during reproductive aging.

### Accelerated Aging and Cellular Senescence in the Irregular Cycling Ovary during Estropausal Transition.

**Fig. 5.**
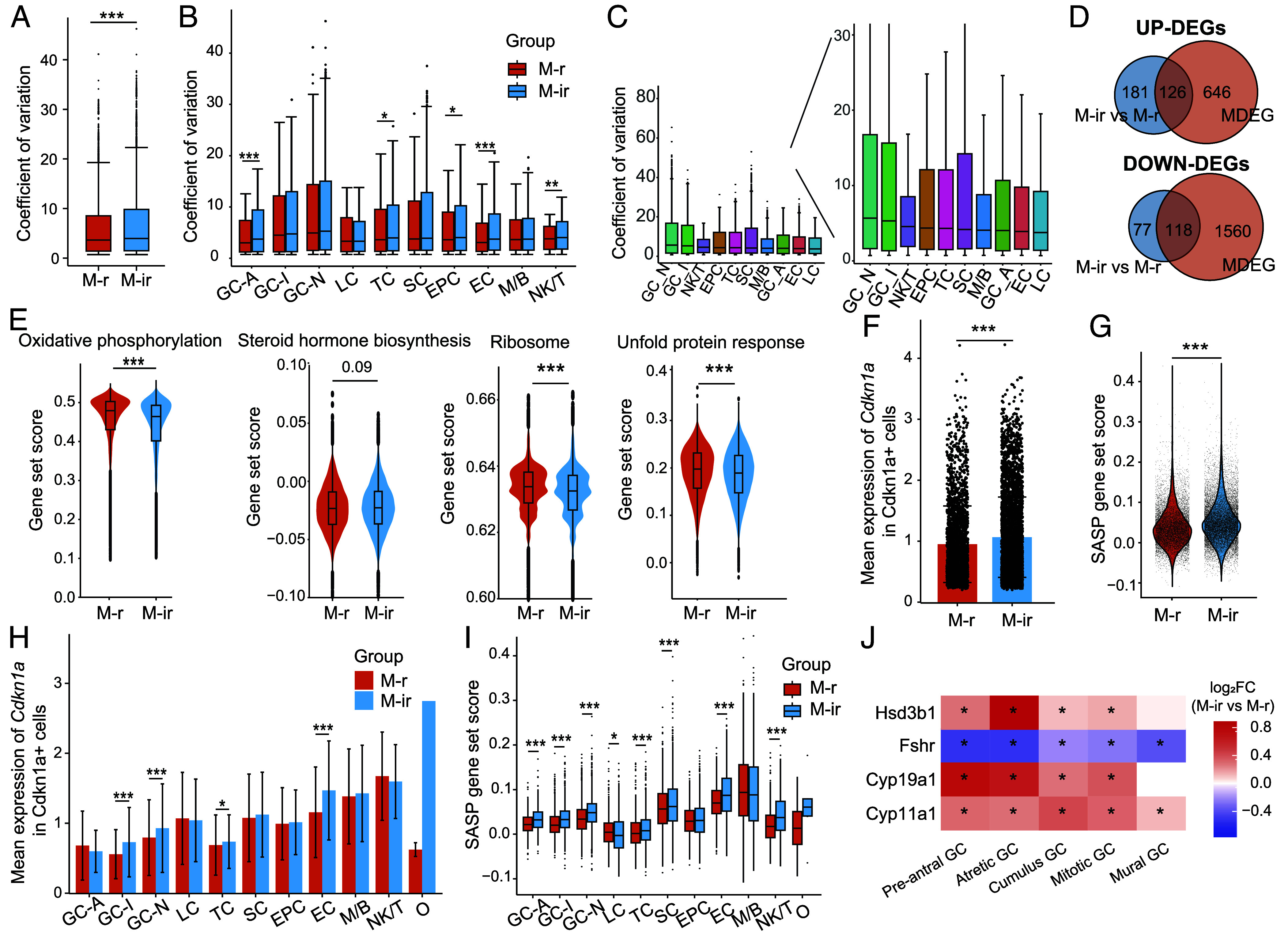
Accelerated ovarian aging and cellular senescence in irregular cycling ovaries during estropausal transition. (*A*) Box plots showing the coefficient variation (CV) of ovarian cells in irregular (M-ir) and regular cycling (M-r) mice at peri-estropause stage (Wilcoxon test, ***Padj < 0.001). (*B*) Box plots showing the CV of each cell type in irregular regular cycling mice at peri-estropause stage (Wilcoxon test, *Padj < 0.05, **Padj < 0.01, ***Padj < 0.001). (*C*) Box plots showing cycle prolongation-associated transcriptional noise examined by CV analysis in each cell type. *Right* panel shows the zoom-in view of the *Left* panel. (*D*) Venn diagrams illustrating the overlap of DEGs between the M-ir vs. M-r comparison and the MDEGs observed during aging. The *Left* panel showing the upregulated DEGs, while *Right* panel showing the downregulated DEGs. (*E*) Box and violin plots showing the gene set score of oxidative phosphorylation, steroid hormone biosynthesis, ribosome, and unfolded protein response in irregular and regular cycling ovary at peri-estropause stage (Wilcoxon test, ***Padj < 0.001). (*F*) Bar plots showing the expression of *Cdkn1a* for the cells that express the gene in irregular and regular cycling ovary at peri-estropause stage (Mean ± SEM, Wilcoxon test, ***Padj < 0.001). (*G*) Violin plots showing the SASP gene set score of ovarian cells at M-r and M-ir group (Wilcoxon test, ***Padj < 0.05). (*H*) Bar plots showing the expression of *Cdkn1a* for the cells that express the gene in each cell type in irregular and regular cycling ovary at peri-estropause stage (Mean ± SEM, Wilcoxon test, ***Padj < 0.001). (*I*) Box plots showing the SASP gene set score of each cell type at M-r and M-ir group (Wilcoxon test, *Padj < 0.05, ***Padj < 0.001). (*J*) Heatmap showing the log_2_ fold change in expression of steroidogenesis-associated genes across granulosa cell subtypes, comparing irregular cycling ovaries to regular cycling counterparts at the peri-estropause stage (Wilcoxon test, *Padj < 0.05).

To unravel the molecular and cellular mechanism that may drive the transition of estropause, we compared the ovaries from irregular cycling mice (M-ir) with regular cycling mice (M-r) during the peri-estropause (M) stage. No significant change was observed in cell composition between M-ir and M-r ovaries (*SI Appendix*, Fig. S10*A*). However, the transcriptional noise was significantly higher in mice with irregular cycle comparing to mice with regular cycle, particularly in *Amh*-high granulosa cells (GC-A), theca cells (TC), epithelial cells (EPC), endothelial cells (EC), and natural killer/T cell types (NK/T) (Fig. 5 *A* and *B*). Granulosa cells (GC) were shown to be the most vulnerable cell types affected by estropausal transition (Fig. 5*C*), which is consistent with the impact of aging (*SI Appendix*, Fig. S1*F*).

Next, we performed DEG analysis and identified 307 upregulated and 195 downregulated DEGs comparing irregular cycling mice with regular cycling mice. Among them, 41% of the upregulated DEGs and 61% of the downregulated DEGs overlapped (Fig. 5*D*) with aging-related upregulated or downregulated MDEGs (*SI Appendix*, Fig. S3*A*). GO analysis indicated the enrichment of DEGs in the same pathways that we found during ovarian aging (Fig. 1*F*), including response to oxidative stress, response to hormone, oxidative phosphorylation, and ribosome across different cell types (*SI Appendix*, Fig. S10*B*). We subsequently calculated pathway scores and found that the changes in pathways in mice with irregular cycles showed consistent trends with those observed during ovarian aging, compared to mice with regular cycles. This included a decrease in oxidative phosphorylation, ribosome, and unfolded protein response pathways, alongside an increase in steroid hormone biosynthesis, in irregular cycling mice compared to regular cycling mice (Fig. 5*E* and *SI Appendix*, Fig. S10 *C*–*F*). Additionally, Cdkn1a expression was significantly elevated in irregular cycling mice compared to regular cycling mice, showing an overall 1.1-fold increase with specific elevations across various cell types: 1.3-fold in GC-I, 1.2-fold in GC-N, 1.1-fold in TC, and 1.3-fold in EC (Fig. 5 *F* and *H*). Moreover, the irregular cycling ovary also showed significantly higher SASP scores, specifically in GC, SC, TC, EC, and NK/T cells (Fig. 5 *G* and *I*). These results indicate that during the estropausal transition, irregularly cycling mice exhibit accelerated ovarian aging signatures and an increased cellular senescence burden compared to regularly cycling counterparts of the same chronological age. Cell type–specific analysis revealed dysregulated hormone synthesis in granulosa cell subtypes, with altered expression of key genes (*Cyp19a1*, *Cyp11a1*, *Fshr*) in irregular cycling mice compared to regular cycling mice, consistent with age-related changes (Fig. 5*J*). In contrast, genes regulating collagen accumulation and hyaluronan metabolism in stromal/theca cells exhibited only minor alterations (*SI Appendix*, Fig. S10 *G* and *H*), suggesting that extracellular matrix remodeling and fibrogenic processes are more closely associated to age-related accumulation rather than the transition to cycle irregularity. Additionally, immune cell composition and function remained relatively stable during this transition, as indicated by unchanged immune cell proportions (*SI Appendix*, Fig. S10*I*) and consistent expression of phagocytosis-related genes (*Mertk* and *Trem2*) in Gpnmb-Mφ (*SI Appendix*, Fig. S10 *J* and *K*). Taken together, our indicated that during peri-estropasue, irregularly cycling ovaries exhibited accelerated aging and cellular senescence features compared with regularly cycling counterparts, suggesting that aging-associated molecular and cellular alterations in the ovarian soma drive the estropausal transition.

## Discussion

This study provides a comprehensive single-cell atlas of the aging mouse ovary across precisely defined reproductive stages, and of ovary-specific senescent cells defined by high SA-β-gal activity. We characterized the transcriptomic dynamics of ovarian aging and delineated the molecular features of ovarian senescent cells. Our analysis reveals that estropausal transition is linked to molecular drivers of aging and cellular senescence in the ovary including increased transcriptional noise, disrupted oxidative phosphorylation and proteostasis, hormone dysregulation in granulosa cells, and elevated expression of the senescence marker *Cdkn1a* and senescence-associated secretory phenotype factors.

The transition from regular cycling to irregular cycling and ultimately to acyclicity, are common features between the preclinical rodent models and women, which provide researchers with opportunities to gain a fundamental understanding of the key elements underlying reproductive aging. The trigger for the transition to menopause remains a topic of debate, with some theories suggesting it could be initiated by a decline in the follicle pool, dysregulation of the hypothalamic-pituitary-gonadal (HPG) axis, or a combination of both (4). Additionally, it was reported that a complex set of changes in neuroendocrine and neurotransmitter signaling involving hypothalamic GnRH neurons and alterations in glutamatergic, GABAergic, and monoaminergic signaling, likely played roles in the early stages of the transition to a reproductively senescent state for rodents, nonhuman primates, and women alike (54, 55). We found that, despite identical genetic background, environment, and chronological age, irregular-cycling mice exhibited accelerated aging and senescence-associated changes in the ovarian somatic cells compared to regular-cycling mice. Our findings suggest that molecular driver of aging and cellular senescence in the ovarian soma could underlie the onset of estropausal transition and reproductive aging. Recent studies further highlight the profound influence of the follicular somatic environment on oocyte quality and reproductive longevity (56, 57). Together, these findings underscore the central role of ovarian somatic cells in reproductive and systemic health, suggesting that targeting their aging-associated molecular programs may help delay estropause (menopause) and reproductive aging.

Senescent cells are typically rare and heterogeneous in vivo, and their identification is complicated by the lack of universal markers. Although cellular senescence has been widely implicated in tissue aging and age-related diseases (14), data on senescent cell accumulation and their functions in the ovary remain limited. Here, we observed an increased senescence burden during ovarian aging and estropausal transition, particularly in granulosa cells. Furthermore, we provide a single-cell transcriptomic profile of live ovarian cells with high SA-β-gal activity and identify ovarian senescence-associated differentially expressed genes (O-SenDEGs) that may serve as potential targets. Granulosa, theca, and stromal cells emerged as the predominant senescent populations. *Cdkn1a* was found as the most consistent senescence-associated cell cycle arrest marker across ovarian cells. However, fewer than 30% of βGal^high^ cells expressed *Cdkn1a*, with proportions varying by cell type, highlighting the heterogeneity of senescence and the challenge of defining senescent cells in vivo (14, 58). βGal^high^ ovarian cells exhibited significantly more pronounced downregulation of oxidative phosphorylation and ribosome pathways than their non-senescent aged counterparts across all ovarian cell types. This pattern is consistent with the transcriptional dysfunction observed during ovarian aging and the estropausal transition; however, the precise causal role of these transcriptional alterations in ovary-specific cellular senescence remains to be further elucidated.

Mitochondrial dysfunction is one of the hallmarks of aging and was suggested to drive both ovarian aging and cellular senescence (59). Abnormalities in mitochondrial ultrastructure and integrity, metabolism, dynamics, and mtDNA mutations and deletions have been associated with the aging of oocytes and granulosa cells (60). In senescent cells, a subset of mitochondria can develop increased outer membrane permeability, allowing mtDNA to leak into the cytoplasm, where it contributes to activation of the SASP (61). The OXPHOS system is central to the cellular energy supply. Inefficiencies in the OXPHOS system result in the production of high levels of ROS, leading to cellular dysfunction and apoptosis (62, 63). The nuclear and mitochondrial genomes each encode different subunits of the electron transfer chain of the OXPHOS system and closely coordinate their activities. In our study, we observed a specific downregulation of mitochondria-encoded, but not nuclear-encoded, OXPHOS subunits in both ovary-specific senescent cells and aged ovarian tissues. This transcriptional imbalance may contribute to the mitochondrial dyshomeostasis observed during ovarian aging and senescence.

Our study highlighted the cell type–specific changes during mouse ovarian aging and estropausal transition. Granulosa cells are critical orchestrators of complex endocrine processes essential for maintaining reproductive function, dynamically producing hormones like estradiol and progesterone, supporting follicular development, and mediating complex hormonal signaling through intricate interactions with gonadotropins such as LH and FSH (64). We observed dysregulation of hormone synthesis–related genes across GC subpopulations during aging, and notably, similar alterations were present in irregular cycling ovaries compared to regular cycling ovaries of the same age, mirroring age-associated molecular changes. These results suggest that disrupted hormone synthesis in GC may contribute to the decline in ovarian function and the onset of estropause. Moreover, GC exhibited the highest vulnerability to increased transcriptional noise and a pronounced rise in senescence markers during both aging and the estropausal transition. Together, these results indicate that accelerated GC aging may be a key driver of reproductive decline, suggesting GC as a potential therapeutic target for mitigating ovarian aging and delaying estropausal transition.

The theca cells that surround growing follicles, along with stromal cells, are critical modulators of the ECM remodeling process during folliculogenesis and ovulation (18). Continuous ECM remodeling throughout reproductive lifespan is thought to contribute to ovarian fibrosis in aged ovaries, driven by increased collagen synthesis and decreased hyaluronan deposition in both mouse and human samples (18, 50). However, recent studies present conflicting findings. Ouni et al. found no age-related differences in collagen or glycosaminoglycans in human ovarian biopsies, despite reduced thick collagen fibers (65). Meanwhile, Winkler et al. observed collagen accumulation in all female reproductive tissues of mice except the ovary and cervix (13). Consistent with a previous single-cell study that compared fibroblasts from 9- vs. 3-mo-old ovaries (12), our analysis showed no changes in collagen gene expressions during aging but noted decreases in collagenase genes, specifically *Mmp2* and *Mmp14*, which may contribute to collagen accumulation. Moreover, changes in hyaluronan metabolism genes during aging may partially explain reduced hyaluronan levels in aged ovaries. However, such aging-associated gene expression alterations in genes involved in collagen and hyaluronan metabolism were not observed during estropausal transition, suggesting that the ECM remodeling and fibrogenic processes are more closely associated with age but not cycle irregularity.

Increased immune cells, especially T cells, is a common age-related change in different aged tissues including the ovary (12, 13, 66). Interestingly, we observed a decrease in phagocytic macrophages (Mφ) and an increase in pro-inflammatory macrophages during ovarian aging. Notably, we identified a unique macrophage subtype with almost no expression of the pro-inflammatory gene *Il1b* and strong expression of the phagocytosis gene *Mertk*, along with high levels of *Atp6v0d2* and *Gpnmb*. *Atp6v0d2* is a macrophage-specific V-ATPase lysosomal subunit that restricts inflammasome activation by facilitating lysosome fusion (67). *Gpnmb* is a phagocytic gene that regulates trafficking of apoptotic debris for degradation and is essential for kidney repair (68). Gpnmb-Mφ can be recruited to injured nerves and mediate efficient myelin clearance via phagocytosis in a sciatic nerve injury model, underscoring their role in tissue damage repair (69). Whether enhancing the clearance capacity of this macrophage subtype could potentially counteract ovarian aging requires further exploration. Additionally, since multinucleated giant cells (MNGCs) are believed to result from the fusion of macrophages with accumulating debris in aged tissues (5), it would be interesting to study if MNGC comes from these Gpnmb-Mφ.

In summary, our study provides a valuable resource for understanding the cellular and molecular basis of ovarian aging and ovary-specific senescent cells, and their association with the estropausal transition in mice. Our findings suggest that aging and cellular senescence-related molecular and cellular alterations in the ovary may drive the estropausal transition and reproductive aging in mice, providing insights into potential biomarkers and therapeutic targets for delaying these processes.

## Materials and Methods

### Experimental Design.

To elucidate the cellular and molecular alterations in the mouse ovary during aging and the estropausal transition, we conducted single-cell RNA sequencing (scRNA-seq) on mouse ovaries from reproductive young (Y, 4.5-mo, n = 5), peri-estropause (M, 10.5-mo, n = 7), and post-estropause (O, 15.5-mo, n = 5) stage. To characterize the features of ovary-specific senescent cells, ovarian cells with high senescence-associated β-galactosidase activity were pooled from five mice (15.5-mo-old) for scRNA-seq. All mice were virgin female C57BL/6 obtained from the Jackson Laboratory. Mice were housed in an SPF environment in groups of up to five per cage under a 12-h light/12-h dark cycle, with ad libitum access to food and water. Colonies were regularly routinely monitored for infections using sentinel mice to ensure health status. All experiments were approved by the ethical committee of Columbia University.

### Estrous Cycle Staging Cytology.

Vaginal smears were collected every day before noon for a minimum of 3 wk using a pipette containing PBS and inserted in the vagina of the restrained mouse. Mucous tissue was then trickled on glass slides and visualized under the microscope to determine the estrous cycle phase (proestrus, estrus, metestrus, and diestrus) according to known cell distribution patterns (70).

### Single-Cell Library Preparation and Data Analysis.

Details of cell dissociation, single-cell library preparation, and data analysis procedures are provided in the *SI Appendix*.

### Analysis of Cellular Senescence, Mitochondrial Function, and Immune Cell Localization in Mouse Ovaries.

Details of SPiDER-β-Gal staining, mitochondrial membrane potential measurement and immunofluorescence staining in mouse ovaries are provided in the *SI Appendix*.

## Supplementary Material

Appendix 01 (PDF)

## Data Availability

Raw data and expression count matrices of 10× scRNA-seq are deposited in GEO (Accession number is GSE267729) (71). Data are also available on CELLxGENE (https://cellxgene.cziscience.com/collections/460007a7-e893-4fc9-8b84-b52679b4561e) (72) where users can browse online or download files compatible with Scanpy and Seurat.
