# Supplementary material for: Single-cell atlas of the mouse ovary reveals molecular drivers of aging and senescence during the estropausal transition
Source: Proc Natl Acad Sci U S A. 2026 Jul 21;123(30):e2600323123. doi: 10.1073/pnas.2600323123 (PMC13416851; doi:10.1073/pnas.2600323123)
Supplement: Supplementary file 1 — Appendix 01 (PDF) [file pnas.2600323123.sapp.pdf]

PNAS

Supporting Information for

Single-cell atlas of the mouse ovary reveals molecular drivers of aging and senescence during the estropausal transition

Xifan Wang[1†*], Jiping Yang[1†], Chen Jin[1], Xizhe Wang[1], Daniela Contreras[1], Melody Devos[1], Maggie M. Kane[2], Michael G. Rosenfeld[3*], Yousin Suh[1, 2*]

[†] These authors contributed equally to this work.
[*] Correspondence: xw2790@cumc.columbia.edu (X.F.W.), mrosenfeld@health.ucsd.edu (M.R.) and ys3214@cumc.columbia.edu (Y.S.)

This PDF file includes:

Supporting text (Materials and Methods)
SI References
Figures S1 to S12
Table S1

Materials and Methods

Cell suspension of mouse ovaries and library preparation for Single-cell RNA-seq

Estrous cycle stages of mice were measured before noon and mice at diestrus stage were anesthetized and perfused with PBS. Both left and right ovaries of each mouse were isolated and immersed in chilled M2 medium (Sigma, M7167) for single-cell suspension preparation, as we observed morphological heterogeneity between the left and right ovaries in some mice, particularly in reproductively aged mice. Surrounding fat and connective tissue were trimmed and removed. Two intact ovaries were minced in 1.5 ml Eppendorf tube and treated by enzymatic digestion in dissociation buffer (M2 medium supplemented with 30 ug/ml Liberase DH (Roche, LIBDH-RO) and 1000U/mL DNase (Thermo Fisher)) at 37°C for 15 min with shaking on thermomixer (1000 rpm). The tube was briefly spun at 1000 rpm and the supernatant was collected in a 15 ml centrifuge tube. Next, the remaining tissues were cut and incubated in dissociation buffer for 10-15 min with shaking. The cell suspension was pooled with the supernatant in a 15 mL tube and neutralized with M2 medium with 1% BSA. The pooled cell suspension was filtered with a 70-um cell strainer (Miltenyi Biotec) and was centrifuged. Cell pellets were incubated with RBC Lysis Buffer (Thermo Fisher) to remove red blood cells and subsequently washed with 1% BSA in PBS and 10% FBS in EMEM. Pelleted cells were resuspended in 10% FBS in EMEM and further submitted to Columbia Genomic Core for scRNA-seq library construction. Briefly, the cell suspension was loaded onto Chromium Single Cell Chip from 10x Genomics Chromium Single Cell 3' Reagent Kits v3, aiming for a target output of 5000 cells per sample. Following reverse transcription, cDNA amplification, indexed adaptor ligation, and library amplification were performed according to the manufacturer's protocol. The final libraries were sequenced on Illumina NovaSeq 6000 at Columbia Genome Center.

Processing and quality control of scRNA-seq data

Raw FASTQ reads were processed using Cellranger analysis pipeline (version 5.0.1) (10× Genomics). Cellranger count command was used to align reads to the mouse genome reference (mm10 - 2020-A) and generate the gene expression matrices. The filtered gene-barcode count matrices were used for quality control with R package Seurat (version 4.1.0)(1). Specifically, quality control filtered out cells with fewer than 200 genes, cells with more than 6000 genes, or cells with more than 15% mitochondrial genes by Seurat "*subset*" function. Additionally, we

identified potential doublets introduced by technical artifact in each sample using DoubletFinder (version 2.0.3)(2) and excluded the doublets by Seurat "*subset*" function. After integration and clustering, one ambient RNA cluster lacking specific gene markers and with low gene content was discarded.

scRNA-seq data analysis and cell-type identification

The R package Seurat (version 4.1.0)(1) was used for scRNA-seq data analysis, including data normalization, dimensionality reduction, cell clustering, and differential expression gene (DEG) analysis. Gene counts were normalized with the LogNormalize method using the Seurat function *"NormalizeData"* and the top 10% highly variable genes (HVGs) identified by the Seurat function *"FindVariableFeatures"* were used for principal component analysis (PCA) using the Seurat function *"RunPCA"*. To remove batch effects, the first 15 PCs were batch corrected using Harmony (version 0.1)(3). To further quantify the batch effects, we evaluated them using complementary approaches, including silhouette scores, iLISI (inverse Local Inverse Simpson's Index), and kBET (k-nearest neighbor batch effect testing). Consistent with the visualization, silhouette scores before (-0.09295207) and after Harmony (-0.09182074) were both close to zero, and quantitative metrics indicated improved local mixing after Harmony integration, with reduced kBET rejection rate (from ~0.329379 to ~0.224479) and increased iLISI scores (from 4.981306 to 5.916409). These results support that batch effects are minimal and that Harmony integration improves local batch mixing without altering the overall structure of the data. Clustering was performed by constructing a K-nearest neighbor (KNN) graph with corrected PCs and applying the Louvain algorithm. Dimensional reduction was performed with Uniform Manifold Approximation and Projection (UMAP) and individual clusters were annotated based on expression of known cell type-specific markers(4-6). Violin/distribution plots per cluster are shown in ***SI Appendix*, Fig. S11 and S12**. Three GC clusters were identified using unbiased, unsupervised clustering of single-cell transcriptomic profiles, free from a priori biological assumptions. Post-hoc annotation mapped these clusters onto established stages of folliculogenesis: the GC-A represents the early gonadotrophin independent stage (Amh); the GC-I captures the intermediate gonadotrophin-sensitive proliferating stage (Inhbb, Mki67, Top2a); and the GC-N aligns with the late mature stage (Nppc, Fshr, Cyp19a1). Cell type-specific markers were identified with the Seurat function *"FindAllMarkers"* for genes detected in at least 25% of cells and a log

fold-change (LogFC) threshold of 0.25 using MAST test (FindAllMarkers results can be accessed from https://doi.org/10.6084/m9.figshare.32051997).

Single-cell RNA-seq of SPiDER-βGal^{high} ovarian cells

Ovaries from 5 female C57BL/6 mice (10 ovaries total) at 15.5-month-old were pooled and processed using the same method as described before to obtain single-cell suspensions. Cells were stained using the Cellular Senescence Detection Kit (SG04, Dojindo) and sorted by flow cytometry (SONY MA900) to enrich for around top 30% of SPiDER-βGal^{high} cells based on fluorescence at GFP channel. The sorted cells were pelleted, resuspended in 10% FBS in EMEM, and submitted to the Columbia Genomics Core for scRNA-seq library preparation using the 10x Genomics Chromium Single Cell 3' Reagent Kits v3, with a target recovery of 5,000 cells per sample. Final libraries were sequenced on the Illumina NovaSeq 6000 platform at the Columbia Genome Center.

Mapping of SPiDER-βGal^{high} single-cell dataset to the reference ovarian atlas

The SPiDER-βGal^{high} single-cell dataset was normalized and mapped to the reference ovarian atlas using the Seurat package. Transfer anchors were identified with FindTransferAnchors using the first 10 dimensions of the reference reduction. Cell type annotations were initially transferred to the query dataset using TransferData to generate predicted labels. The reference UMAP model was computed with RunUMAP and retained, and query cells were projected into the reference embedding using MapQuery, integrating the transferred labels with the reference UMAP. Predicted cell identities were stored as metadata and visualized using DimPlot.

Differentially expressed gene (DEG) analysis

Cell-type specific pairwise differential expression analysis was performed with the Seurat function "*FindMarkers*" to identify DEGs between Y and M, between Y and O, and between M_ir and M_r in each cell type. The log fold change and adjusted P-value of each DEG were calculated by the MAST test and only those expressed in at least 25% cells with $|avg\_log_2FC| > 0.25$ and "pval_adj" < 0.05 were considered to be DEGs. Gene ontology analysis was performed using Metascape(7).

Cell-type specific monotonic DEGs (MDEGs) analysis was performed by following steps: 1) Cell type–specific differential expression analyses were performed for each age group (Y, M, and O)

versus the remaining cells using Seurat (FindMarkers, MAST test; min.pct = 0.25, $|\log_2 FC| > 0.25$, adjusted $P < 0.05$). Genes identified as significant in any comparison were merged to generate a comprehensive set of DEGs for each cell type; 2) gene expression of these whole DEGs were z scored and locally estimated scatterplot smoothing (LOESS) regression was fitted for each gene to obtain predicted age-based gene expression values; 3) genes were further categorized into four trajectory patterns as monotonic up (MU), up and down (UD), down and up (DU) and monotonic down (MD) according to their changes from Y to M to O. Monotonic DEGs (MDEGs) were defined as genes belonging to the MU or MD categories.

Senescence-associated differential expression analysis was performed using the Seurat function "FindMarkers". Within each identified cell type, DEGs were calculated between the isolated SPiDER-βGal^{high} cells and all cells derived from 15.5-month irregular cycling ovaries. The log fold change and adjusted P-value of each DEG were calculated by the MAST test and only those expressed in at least 25% cells with $|\text{avg\_log}_2\text{FC}| > 0.25$ and "pval_adj" < 0.05 were considered to be DEGs.

Gene set score

Gene set scores of cell identity, senescence-associated secretory phenotype (SASP), unfolded protein response, mitochondria-encoded genes, nuclear-encoded OXPHOS components, NF-kB signaling pathway, DNA repair response, collagen-associated genes, M1 pro-inflammation signature, and M2 Anti-inflammation signature, were evaluated by the Seurat function *"AddModuleScore"* with corresponding gene lists, respectively. Gene lists were shown in the **Supplementary Table 1.**

Coefficient of variation analysis between different groups

Analysis of age-relevant coefficient of variation (CV) was used to observe the aging effects on different cell types as described previously(8, 9). The Seurat "*FindVariableFeatures*" function was used to identify highly variable genes (HVGs). The top 10% variable genes (2744 of 27,445 genes) were selected for downstream analysis. Next, the absolute value of the cell-paired-distance $d_{c,x}$ was calculated for each HVG x between all cells from two different groups in each cell type c.

To compare between the young group (Y, cell number = y) and the post-estropause group (O, cell number = o), the formula is:

$$d_{c,x} = |X_{c,i} - X_{c,j}|; i \in \{1, 2, \dots, y\}d, j \in \{1,2, \dots, o\}$$

To compare between the young group (Y, cell number = y) and the peri-estropause group (O, cell number = m), the formula is:

$$d_{c,x} = |X_{c,i} - X_{c,j}|; i \in \{1, 2, \dots, y\}, j \in \{1,2, \dots, m\}$$

To compare between regular cycling group (M-r, cell number = r) and irregular cycling group (M-ir, cell number = ir) during peri-estropause stage, the formula is:

$$d_{c,x} = |X_{c,i} - X_{c,j}|; i \in \{1, 2, \dots, r\}, j \in \{1,2, \dots, ir\}$$

Next, the arithmetic mean of $d_{c,x}$ ($\mu_{c,x}$) and the standard deviation of $d_{c,x}$ ($\sigma_{c,x}$) were calculated. Accordingly, the transcriptional variation of HVG x in each cell type c between two different groups is defined by the following formula:

$$CV_{c,x} = \frac{\sigma_{c,x}}{\mu_{c,x}}$$

Transcriptional noise analysis

The Seurat "*FindVariableFeatures*" function was used to identify highly variable genes (HVGs). The top 10% variable genes (2744 of 27,445 genes) were selected for downstream analysis. The absolute value of the cell-paired-distance $d_{c,x}$ was calculated for each HVG x within each cell type c (cell number = n), the formula is:

$$d_{c,x} = |X_{c,i} - X_{c,i}|; i \in \{1, 2, , n\}$$

Next, the arithmetic mean of $d_{c,x}$ ($\mu_{c,x}$) and the standard deviation of $d_{c,x}$ ($\sigma_{c,x}$) were calculated. Accordingly, the transcriptional noise of HVG x in each cell type c in a specific group is defined by the following formula:

$$CV_{c,x} = \frac{\sigma_{c,x}}{\mu_{c,x}}$$

Pairwise cosine similarity

For mouse ovary, the middle aged and reproductively old ovary were compared to the reproductively young ovary to identify the aging-associated DEGs. For other mouse tissues, the

data were obtained from Tabula Muris Senis(10), using the 3-month group as a young reference, and compared it with both early-aged (18-month) and late-aged (24- or 30 month) groups. The analytical approach of DEGs in mouse ovaries were utilized to data of other mouse tissues. The log2(fold changes) of aging-associated DEGs in each cell type were extracted. Pairwise cosine similarity between all cell types within each tissue was computed using the function "*cosine*" in lsa package (v0.73.2)(11). This generated a similarity matrix per tissue. The mean cosine similarity for other mouse tissue was statistically compared to the mean similarity in mouse ovary cell types using a two-sided Wilcoxon rank sum test.

Gene set variation analysis (GSVA)

R package GSVA (v1.44.5)(12) was utilized to estimate pathway activity scores for each cell, based on KEGG pathways in the MSigDB database (R package msigdbr v7.5.1). The GSVA score was normalized with the z-score method and used to indicate the pathway activity including ribosome, steroid hormone biosynthesis, and oxidative phosphorylation.

Trajectory Analysis

The R package Monocle 3 (version 1.3.1)(13) package was applied to construct pseudotime trajectories and to identify genes that play key roles during cell state transition in granulosa cells (GC), stromal and theca cells (SCTC). The original embedding of UMAP reduction was used. Monocle algorithm learns the sequence of gene expression changes each cell must go through as part of a dynamic biological process and places each cell at its proper position in the trajectory. The construction of single-cell trajectories was performed using default parameters. After learning the trajectory graph, the pre-antral granulosa cells in the GC subset and the interstitial stromal cells in the SCTC subset were chosen as the root node to order cells along the trajectory. Significantly changed genes along the pseudotime trajectory were identified using the Moncle3 graph-autocorrelation analysis to find genes that vary over the trajectory.

RNA velocity analysis

To perform the RNA velocity analysis, we rerun the Cellranger count command with the argument --include-introns true. The aligned bam files (possorted_genome_bam.bam) generated by Cellranger were used to count the spliced reads and unspliced reads by the velocity (version

0.17.17)(14). The RNA velocity estimation and visualization as streamlines were done by using the scvelo (version 0.3.1).

SEVtras analysis

The secretion of small extracellular vesicles was analyzed using SEVtras (version 0.2.8, small extracellular vesicles(sEV)-containing droplet identification in scRNA-seq data)(15). The raw_feature_bc_matrix folder generated by the Cellranger count command was used as input in *SEVtras.sEV_recognizer* function with default parameters to identify sEV-containing droplets. With the output of SEVtras.sEV_recognizer, sample- or cell type-specific sEV secretion activity indexes (ESAI) were calculated using *SEVtras.ESAI_calculator* function with default parameters.

Cell-cell communication

Cell-cell communication analysis was performed using the R package CellChat (Version 1.5.0)(16). based on the expression of known ligand-receptor pairs in different cell types. Mouse ligand-receptor interaction database (CellChatDB.mouse) were used to identify over-expressed ligands or receptors in each cell type. Cell-cell communication network was inferred based on the over-expressed ligands-receptors interactions if either ligand or receptor are over-expressed. Significant cell-cell communication was inferred by assigning each interaction with a probability value and performing a permutation test.

SPiDER-β-Gal staining

Cellular Senescence Detection Kit (SG04, Dojindo) was used to detect SA-β-Gal activity according to the manufacturer's manual. For analysis by flow cytometry, mouse ovaries were firstly dissociated into single-cell suspension (see details in ***SI Appendix***). Cells were incubated with Bafilomycin A1 working solution in an M2 medium at 37 ºC for 1 hour to inhibit endogenous β-galactosidase activity. The SPiDER-β-Gal working solution was further added to stain cells at 37 ºC for 30 minutes. Cells were washed with M2 medium and analyzed by BD LSR flow cytometer. We calculated the background-removed SPiDER-β-Gal intensity for each sample by subtracting the mean GFP intensity in the unstained sample from the mean GFP intensity in the stained sample. Subsequently, these background-removed intensities were normalized to the young group.

Mitochondrial membrane potential measurement

Mitochondrial Membrane Potential Detection Kit (MT13-10, Dojindo) was used to detect Mitochondrial membrane potential in ovarian cells according to the manufacturer's manual. Mouse ovaries were processed using the same method for single-cell RNA-seq to obtain single-cell suspension. Cells were incubated with MT-1 Dye in M2 medium at 37 ºC for 30 minutes and washed with M2 medium. Cells were resuspended in 1x Imaging Buffer solution and analyzed by BD LSR flow cytometer. Unstained samples were utilized to establish the positive cutoff on the PE channel and the mean intensity of PE-positive cells in stained sample was calculated to represent the mitochondrial membrane potential of each sample. Subsequently, the intensities were normalized to the young group.

Immunofluorescence staining

Ovaries were freshly collected from mice at diestrus, fixed in 4% paraformaldehyde, embedded in Tissue-Tek O.C.T. Compound (Sakura Finetek). and sectioned at 10 µm thickness. Tissue sections were permeabilized in 0.04% Triton X-100/PBS for 15 mins. After blocking with 10% donkey serum (Jackson ImmunoResearch Labs)/PBS for 1 h, sections were incubated with primary antibodies at 4 °C overnight and the corresponding secondary antibody (Invitrogen) at RT for 45 mins, slides were mounted using ProLong Gold Antifade Mountant (Thermo Fisher). Nuclei were stained with Hoechst 33342 (62249, Thermo Fisher). Primary antibodies used in this study were listed below: anti-Gpnmb (R&D, AF2330), and anti-Cd68 (Abcam, ab53444). Images were captured using the Leica Stellaris 8 Confocal Microscope.

Reference

1. Y. Hao *et al.*, Integrated analysis of multimodal single-cell data. *Cell* **184**, 3573-3587 e3529 (2021).
2. C. S. McGinnis, L. M. Murrow, Z. J. Gartner, DoubletFinder: Doublet Detection in Single-Cell RNA Sequencing Data Using Artificial Nearest Neighbors. *Cell Syst* **8**, 329-337 e324 (2019).
3. I. Korsunsky *et al.*, Fast, sensitive and accurate integration of single-cell data with Harmony. *Nat Methods* **16**, 1289-1296 (2019).
4. M. E. Morris *et al.*, A single-cell atlas of the cycling murine ovary. *Elife* **11** (2022).
5. J. V. V. Isola *et al.*, A single-cell atlas of the aging mouse ovary. *Nat Aging* **4**, 145-162 (2024).
6. L. Zhang *et al.*, Single-Cell Analyses Inform Mechanisms of Myeloid-Targeted Therapies in Colon Cancer. *Cell* **181**, 442-459 e429 (2020).
7. Y. Zhou *et al.*, Metascape provides a biologist-oriented resource for the analysis of systems-level datasets. *Nat Commun* **10**, 1523 (2019).
8. M. C. Salzer *et al.*, Identity Noise and Adipogenic Traits Characterize Dermal Fibroblast Aging. *Cell* **175**, 1575-1590 e1522 (2018).
9. S. Wang *et al.*, Single-Cell Transcriptomic Atlas of Primate Ovarian Aging. *Cell* **180**, 585-600 e519 (2020).
10. C. Tabula Muris, A single-cell transcriptomic atlas characterizes ageing tissues in the mouse. *Nature* **583**, 590-595 (2020).
11. C. Jin *et al.*, Molecular and genetic insights into human ovarian aging from single-nuclei multi-omics analyses. *Nature Aging* 10.1038/s43587-024-00762-5 (2024).
12. S. Hanzelmann, R. Castelo, J. Guinney, GSVA: gene set variation analysis for microarray and RNA-seq data. *BMC Bioinformatics* **14**, 7 (2013).
13. J. Cao *et al.*, The single-cell transcriptional landscape of mammalian organogenesis. *Nature* **566**, 496-502 (2019).
14. G. La Manno *et al.*, RNA velocity of single cells. *Nature* **560**, 494-498 (2018).
15. R. He, J. Zhu, P. Ji, F. Zhao, SEVtras delineates small extracellular vesicles at droplet resolution from single-cell transcriptomes. *Nat Methods* **21**, 259-266 (2024).
16. S. Jin *et al.*, Inference and analysis of cell-cell communication using CellChat. *Nat Commun* **12**, 1088 (2021).

Supplementary figures

Figure S1

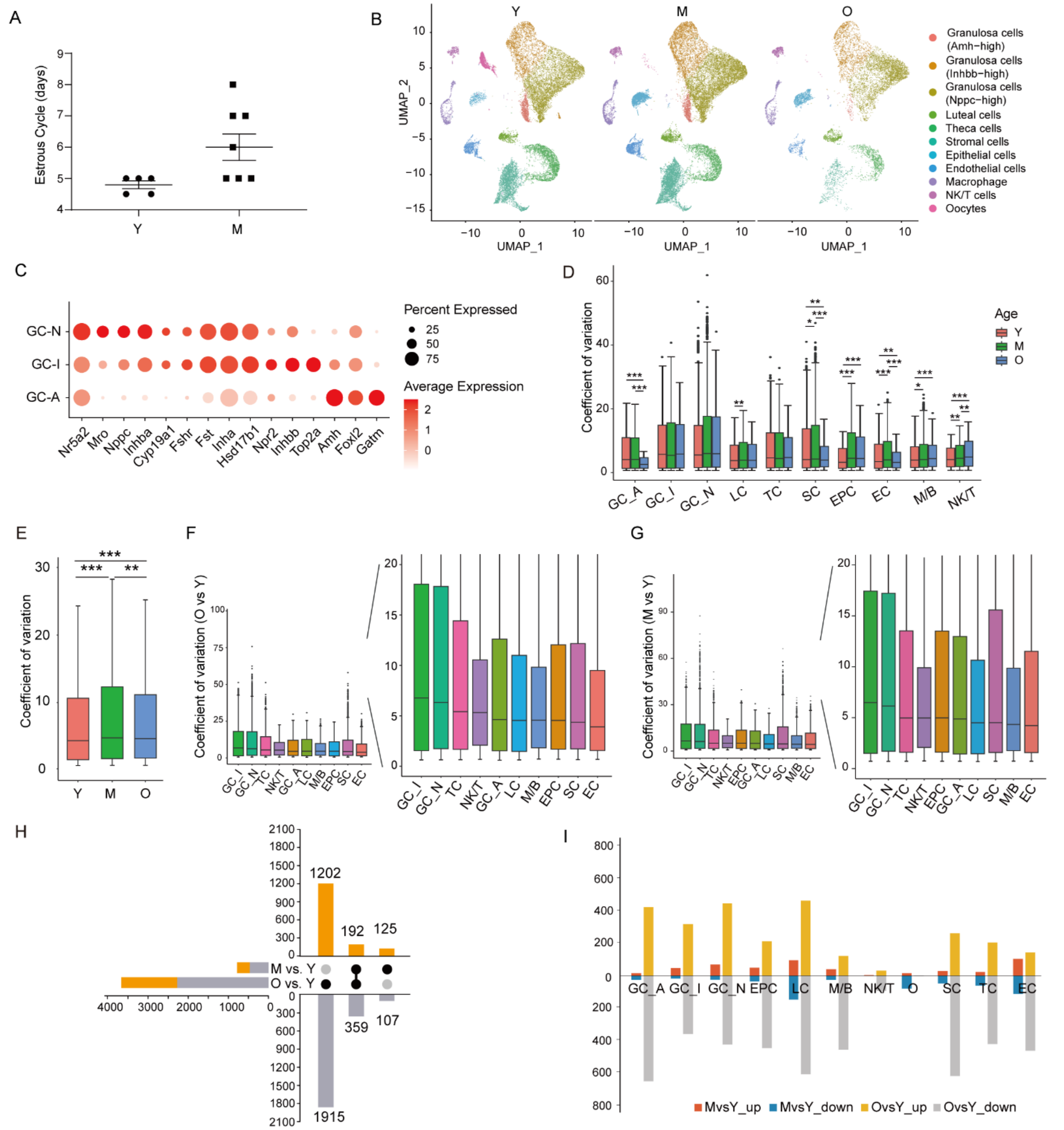

Fig. S1. Global transcriptional change during mouse ovarian aging across reproductive lifespan. (A) Scatter plots showing the estrous cycle length of each young (Y) and peri-estropause (M) ovary submitted for single-cell sequencing. Post-estropause (O) ovaries submitted for scRNA-seq were acyclic. **(B)** Uniform manifold approximation and projection (UMAP) plots showing the cell types of young (Y), peri-estropause (M) and post-estropause (O) mouse ovaries. **(C)** Dot plots showing the expression of representative genes for granulosa cells (GC). GC-A, Amh-high granulosa cell; GC-I, Inhbb-high granulosa cell; GC-N, Nppc-high granulosa cell. **(D)** Box plots showing the coefficient variation (CV) of each cell type at each age. Box shows the median and

the quartile range (25%–75%) and the length of whiskers represents 1.5× the IQR. (Wilcoxon test; * Padj < 0.05; ** Padj < 0.01; *** Padj < 0.001). (**E**) Box plots showing the coefficient variation (CV) of ovarian cells at each age group. Box shows the median and the quartile range (25%–75%) and the length of whiskers represents 1.5× the IQR. (Wilcoxon test, ** Padj < 0.01, *** Padj < 0.001). (**F**) Box plots showing aging-associated transcriptional noise examined by CV analysis (O versus Y) in each cell type. Right shows the zoom-in view of the left panel. **(G)** Box plots showing aging-associated transcriptional noise examined by CV analysis (M vs Y) in each cell type. Right shows the zoom-in view of the left panel. **(H)** Upset plots showing the numbers of unique and shared pairwise differentially expressed gene (DEGs) between reproductive young mice and mice of other ages. **(I)** Bar plots showing the numbers of pairwise differentially expressed genes (DEGs) in each cell type between reproductive young mice and mice of other ages.

Figure S2

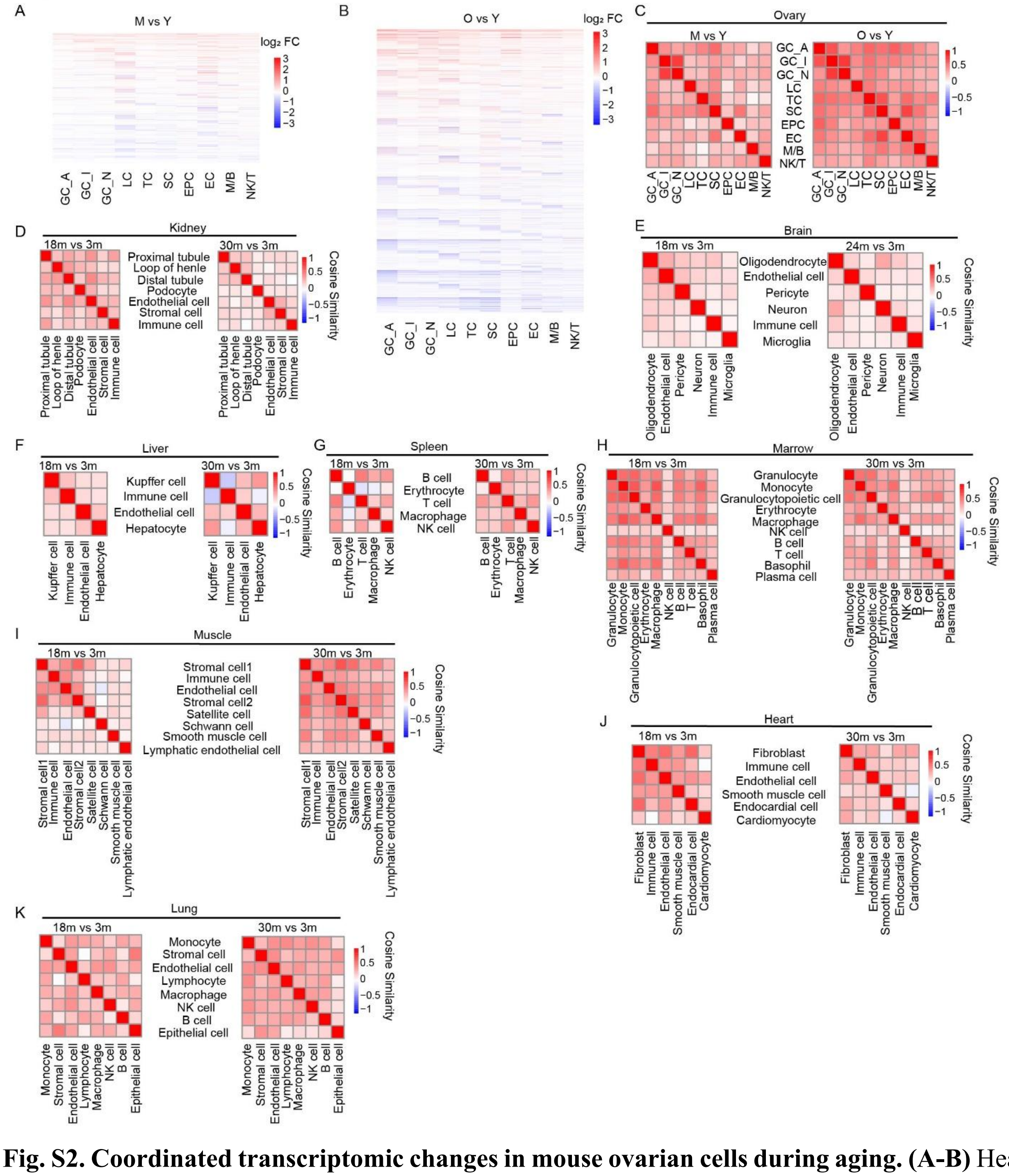

Fig. S2. Coordinated transcriptomic changes in mouse ovarian cells during aging. (A-B) Heat maps showing $\log_2$ fold changes in gene expression of pairwise DEGs in peri-estropause ovaries **(A,** M vs Y**)** and post-estropause ovaries **(B,** O vs Y**)** comparing to young ovaries across different cell types. **(C-K)** Heat maps showing the pairwise cosine similarities of transcriptomic changes during aging between cell types in the mouse ovary **(C)** and brain **(D)**, kidney **(E)**, liver **(F)**, spleen **(G)**, marrow **(H)**, muscle **(I)**, and heart **(J)**, lung **(K)**.

Figure S3

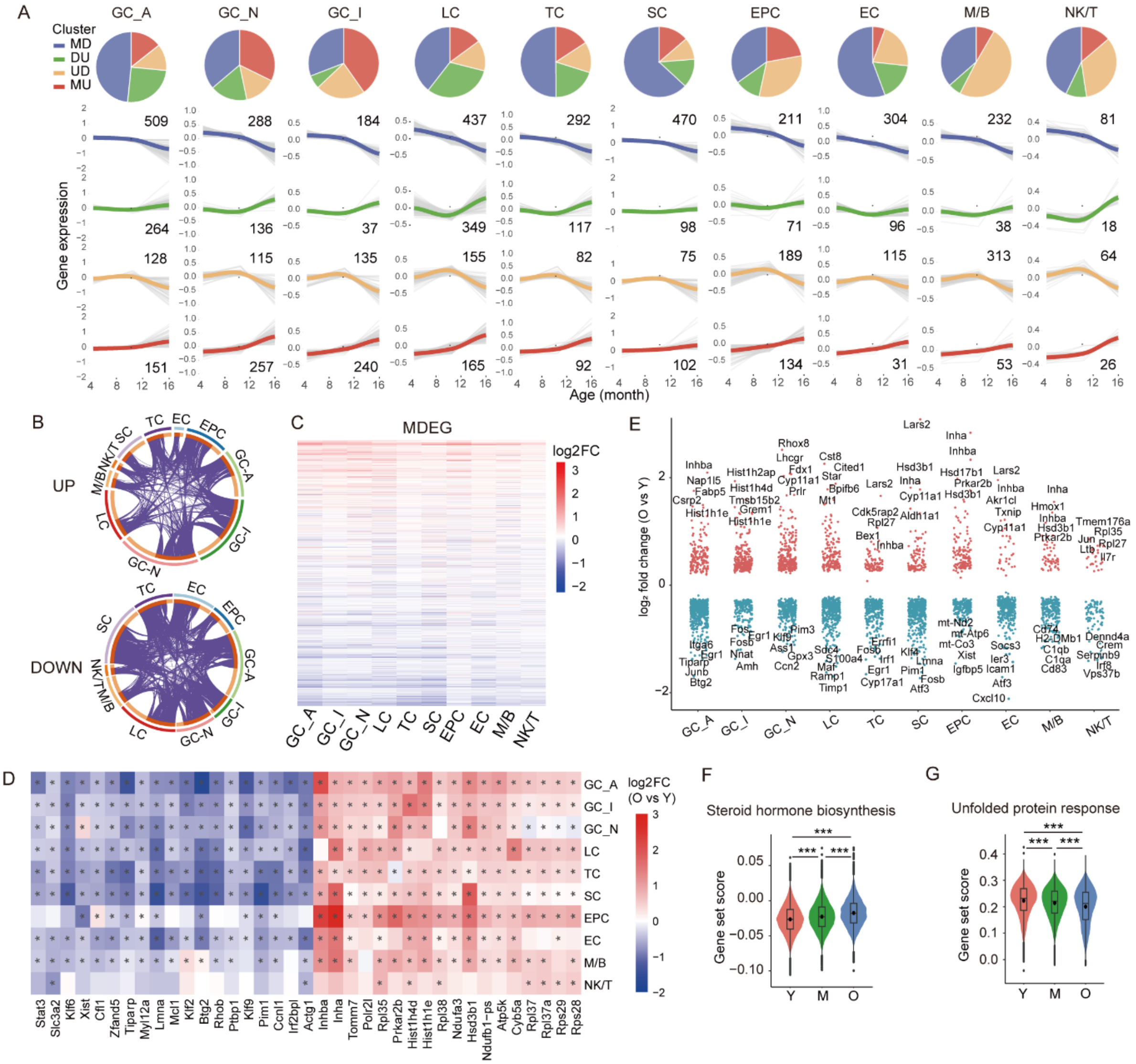

Fig. S3. Gene signatures of mouse ovarian aging. (A) Pie chart showing the proportion of temporal DEGs with four different expression patterns during mouse ovarian aging (MD, monotonic down; DU, down and up; UD, up and down; MU, monotonic up) in each cell type (up panel). Gene trajectories during mouse ovarian aging and the number of genes within each expression pattern in each cell type (down panel). Gene expression was z scored, and trajectories of all genes were estimated by LOESS. **(B)** Circos plots depicting the overlaps among gene lists of ovarian upregulated MDEGs (left) or downregulated MDEGs (right) for each cell type during mouse ovarian aging. The inner circle represents gene lists, and purple curves link identical genes. The genes that hit multiple lists are colored in dark orange, and genes unique to a list are shown in light orange. **(C)** Heat map showing $\log_2$ fold changes (O vs Y) in gene expression of MDEG during mouse ovarian aging in each cell type. **(D)** Heatmap showing the $\log_2$ fold changes in gene expression (O vs Y) of monotonic upregulated DEGs shared by at least 6 cell types and monotonic downregulated DEGs shared by at least 7 cell types. * Indicates a statistically significant difference (Padj < 0.05). **(E)** Strip plots showing $\log_2$ fold change (O vs Y) of monotonic DEGs in each cell

type. Top five upregulated and top five downregulated genes per cluster were labeled. **(F-G)** Violin plots and box plots showing the gene set score of steroid hormone biosynthesis **(F)** and unfolded protein response **(G)** during mouse ovarian aging. Dot mark indicating the mean. Wilcoxon test; *** Padj < 0.001.

Figure S4

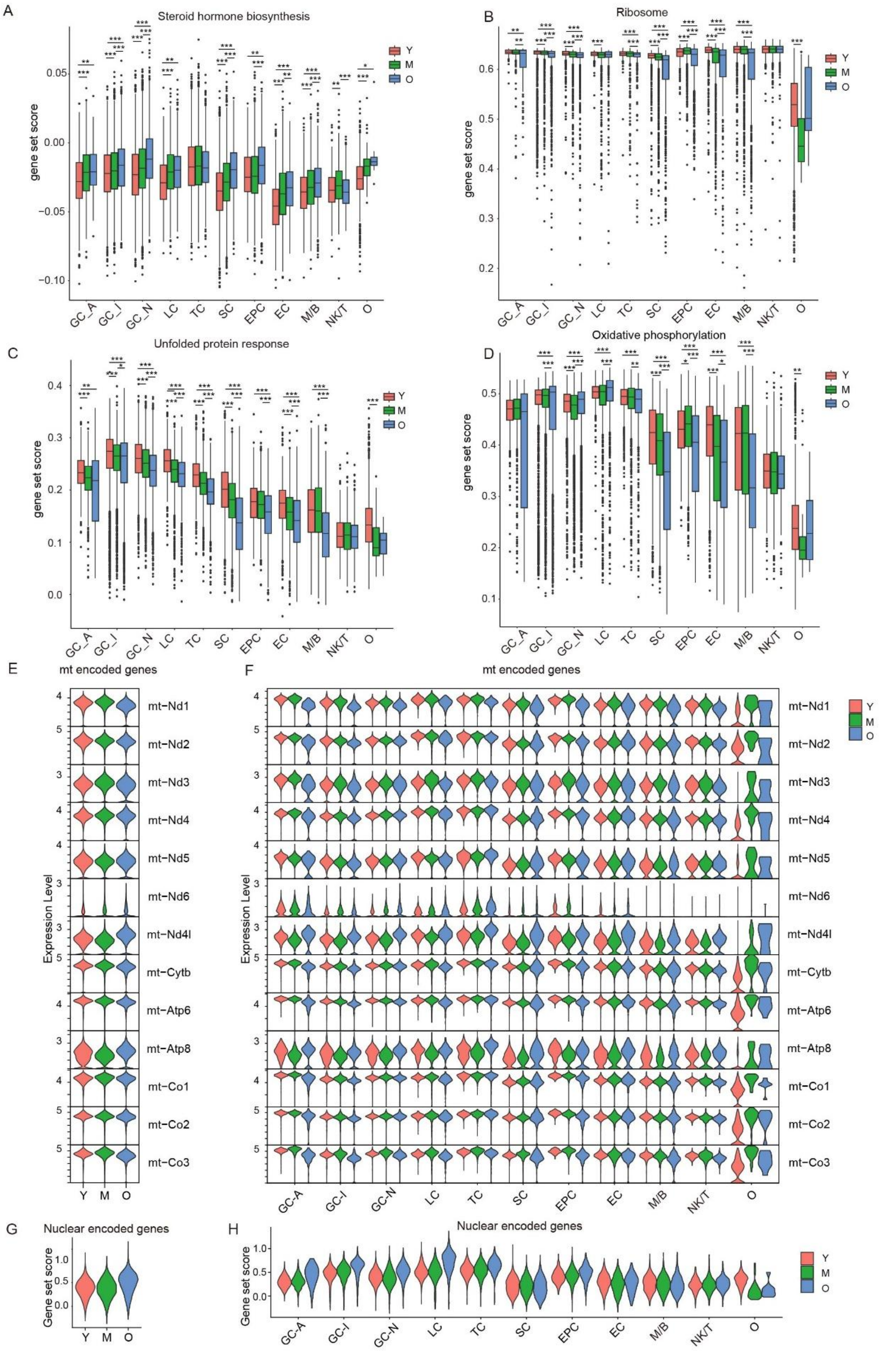
A
Steroid hormone biosynthesis
gene set score
B
Ribosome
C
Unfolded protein response
D
Oxidative phosphorylation
Y
M
O
GC_A
GC_I
GC_N
LC
TC
SC
EPC
EC
M/B
NK/T
E
mt encoded genes
F
mt encoded genes
Expression Level
mt-Nd1
mt-Nd2
mt-Nd3
mt-Nd4
mt-Nd5
mt-Nd6
mt-Nd4l
mt-Cytb
mt-Atp6
mt-Atp8
mt-Co1
mt-Co2
mt-Co3
GC-A
GC-I
GC-N
G
Nuclear encoded genes
Gene set score
H
Nuclear encoded genes

Fig. S4. Gene set scores of aging hallmarks related pathways in each cell type during mouse ovarian aging. (A-D) Box plots showing gene set scores of steroid hormone biosynthesis **(A)**, ribosome **(B)**, unfolded protein response **(C)** and oxidative phosphorylation **(D)** in each cell type at each age (Wilcoxon test; * Padj < 0.05, ** Padj < 0.01, *** P < 0.001). **(E-F)** Violin plots showing expression level of mitochondria encoded genes of electron transport chain in all cell types **(E)** and in each cell type **(F)** at different ages. **(G-H)** Violin plots showing the gene set score of nuclear encoded genes of electron transport chain in all cell types **(G)** and in each cell type **(H)** at different ages.

Figure S5

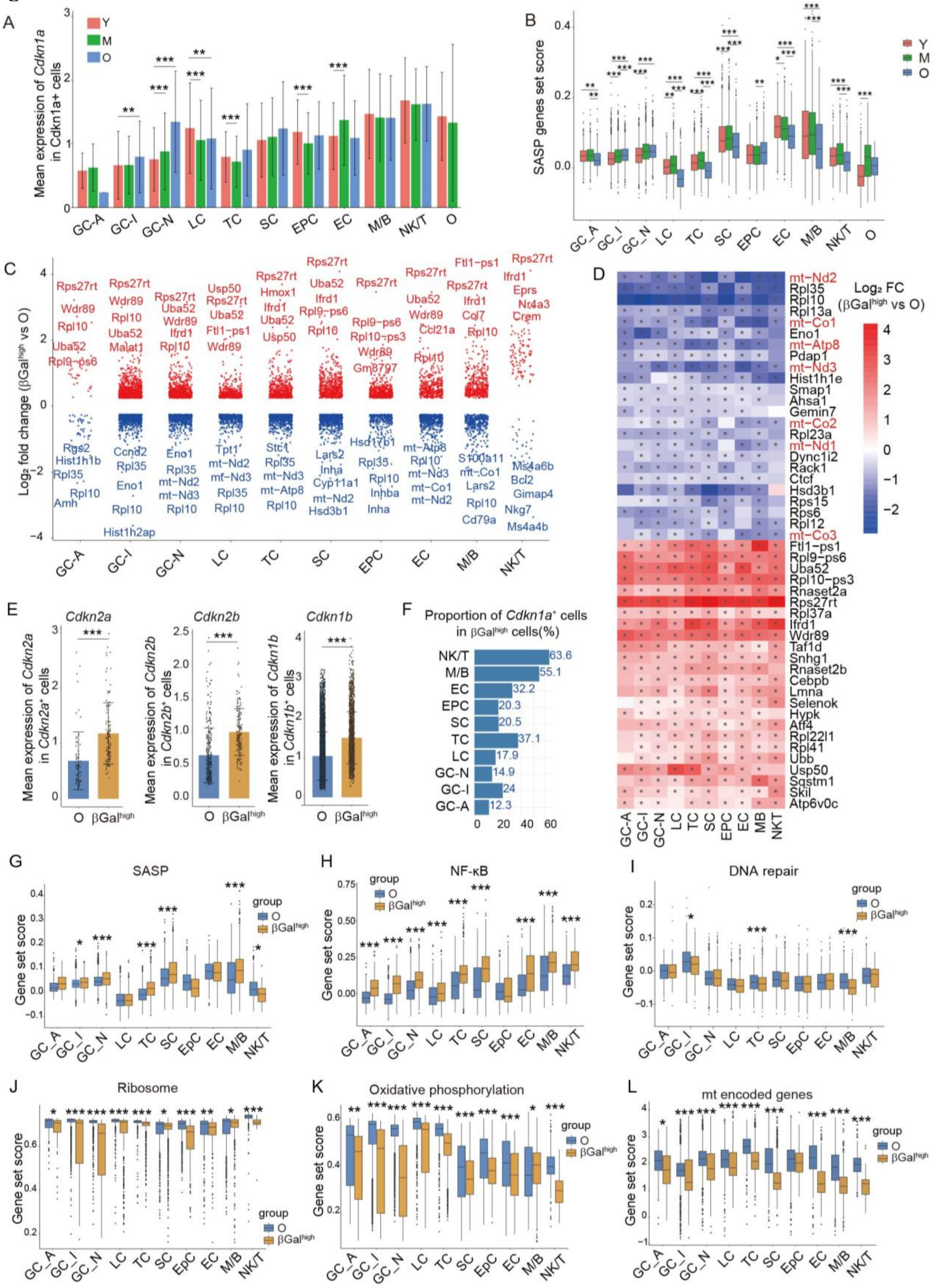

A
Mean expression of Cdkn1a in Cdkn1a+ cells
Y
M
O
GC-A
GC-I
GC-N
LC
TC
SC
EPC
EC
M/B
NK/T
O
B
SASP genes set score
C
Log2 fold change (βGal^high vs O)
D
Log2 FC (βGal^high vs O)
E
Cdkn2a
Cdkn2b
Cdkn1b
Mean expression of Cdkn2a in Cdkn2a+ cells
Mean expression of Cdkn2b in Cdkn2b+ cells
Mean expression of Cdkn1b in Cdkn1b+ cells
O
βGal^high
F
Proportion of Cdkn1a+ cells in βGal^high cells(%)
NK/T 63.6
M/B 55.1
EC 32.2
EPC 20.3
SC 20.5
TC 37.1
LC 17.9
GC-N 14.9
GC-I 24
GC-A 12.3
G
SASP
H
NF-κB
I
DNA repair
J
Ribosome
K
Oxidative phosphorylation
L
mt encoded genes
group
Gene set score

Fig. S5. Transcriptomic signatures of ovary-specific senescent cells. (A) Bar plots showing the expression of *Cdkn1a* in each cell type for the cells that express the gene at each age group. (Mean ± SEM, Wilcoxon test, ** Padj < 0.01, *** Padj < 0.001). **(B)** Box plots showing SASP gene set score in each cell type at each age group (Wilcoxon test; * Padj < 0.05, ** Padj < 0.01, *** Padj < 0.001). **(C)** Strip plots showing $\log_2$ fold change of DEGs between β-gal^{high} cells and age-matched ovarian cells (O) in each cell type. Top five upregulated and top five downregulated genes per cluster were labeled. **(D)** Heatmap showing $\log_2$ fold changes of downregulated DEGs shared across at least 8 cell types and upregulated DEGs shared across at least 9 cell types, comparing β-gal^{high} cells to age-matched ovarian cells (O) * Indicates a statistically significant difference (Padj < 0.05). **(E)** Bar plots showing the expression of *Cdkn2a, Cdkn2b and Cdkn1b* for the cells that express the gene in β-gal^{high} cells and age-matched ovarian cells (O). (Mean ± SEM, Wilcoxon test, *** Padj < 0.001). **(F)** the proportion of β-gal^{high} cells expressing *Cdkn1a* in different cell types. **(G-L)** Box plots showing the gene set scores of SASP **(G)**, NK-κB pathway **(H)**, DNA repair **(I)**, ribosome **(J)**, oxidative phosphorylation **(K)**, and mitochondrial (mt)-encoded genes **(L)** in β-gal^{high} cells and age-matched ovarian cells (O) across cell types (Wilcoxon test; * Padj < 0.05, ** Padj < 0.01, *** Padj < 0.001).

Figure S6

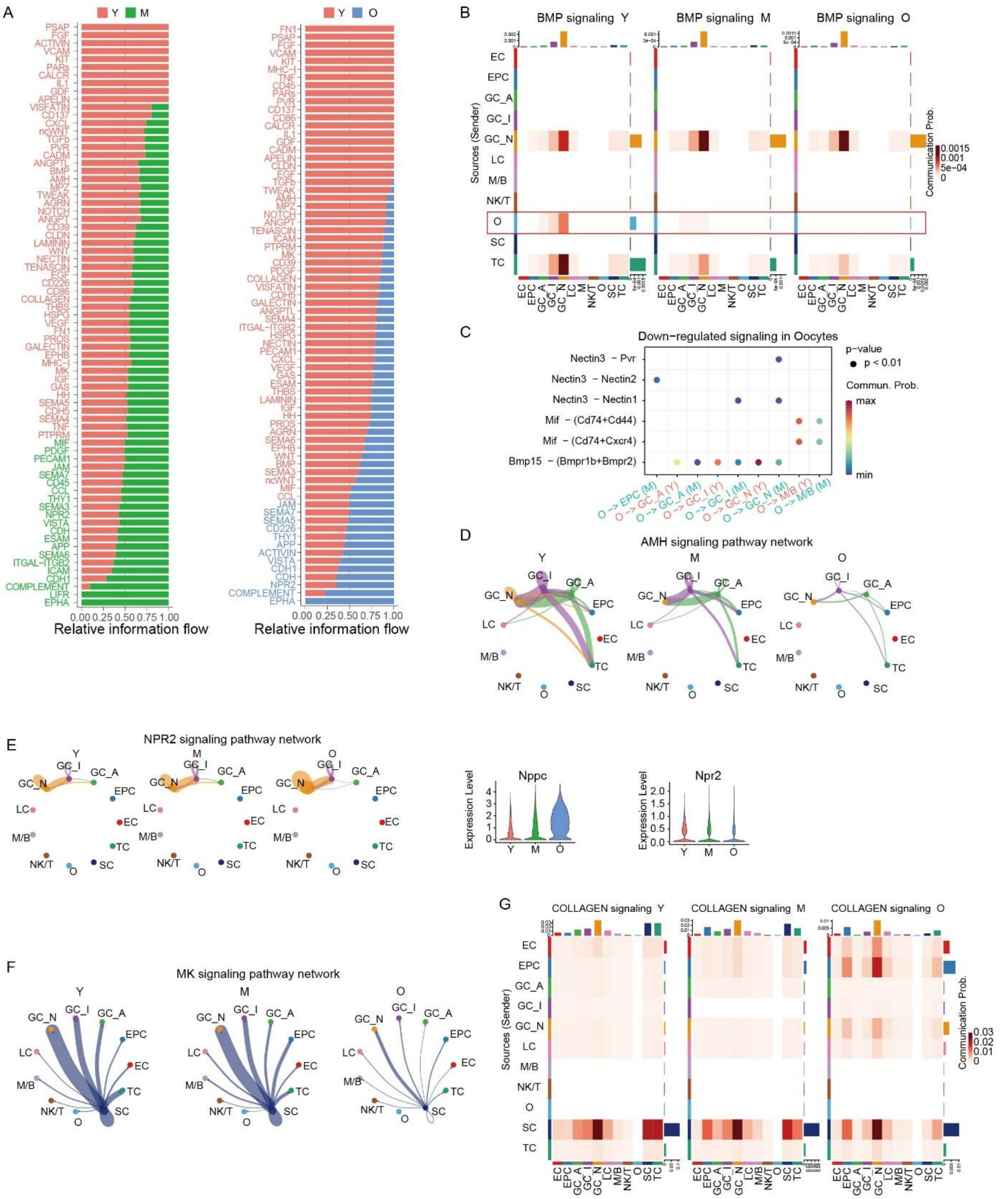

Fig. S6. Change of cellular interactions among different cell types during mouse ovarian aging. (A) Stacked bar charts showing the significantly changed signaling information flow between peri-estropause ovaries (M, left panel) or post-estropause ovaries (O, right panel) compared with young ovaries. The top signaling pathways colored red are enriched in young (Y) ovaries, and these colored greens are enriched in peri-estropause (M) or post-estropause (O)

ovaries. **(B)** Heat map showing BMP signaling network in young (Y), peri-estropause (M), and post-estropause (O) ovaries. Rows and columns represent sources and targets, respectively. Bar plots on the right and top represent the total outgoing and incoming interaction scores respectively. **(C)** bubble plots showing the significantly downregulated signaling (ligand-receptor pairs) in oocytes during ovarian aging. **(D)** Circle plots showing AMH signaling network in young (Y), peri-estropause (M), and post-estropause (O) ovaries. **(E)** Circle plots showing NPR2 signaling network in young (Y), peri-estropause (M), and post-estropause (O) ovaries (left panel). Violin plots showing the expression of ligand and receptor of NPR2 signaling at each age group (right panel). **(F)** Circle plots showing MK signaling network in young (Y), peri-estropause (M), and post-estropause (O) ovaries. **(G)** Heat map showing COLLAGEN signaling network in young (Y), peri-estropause (M), and post-estropause (O) ovaries.

Figure S7

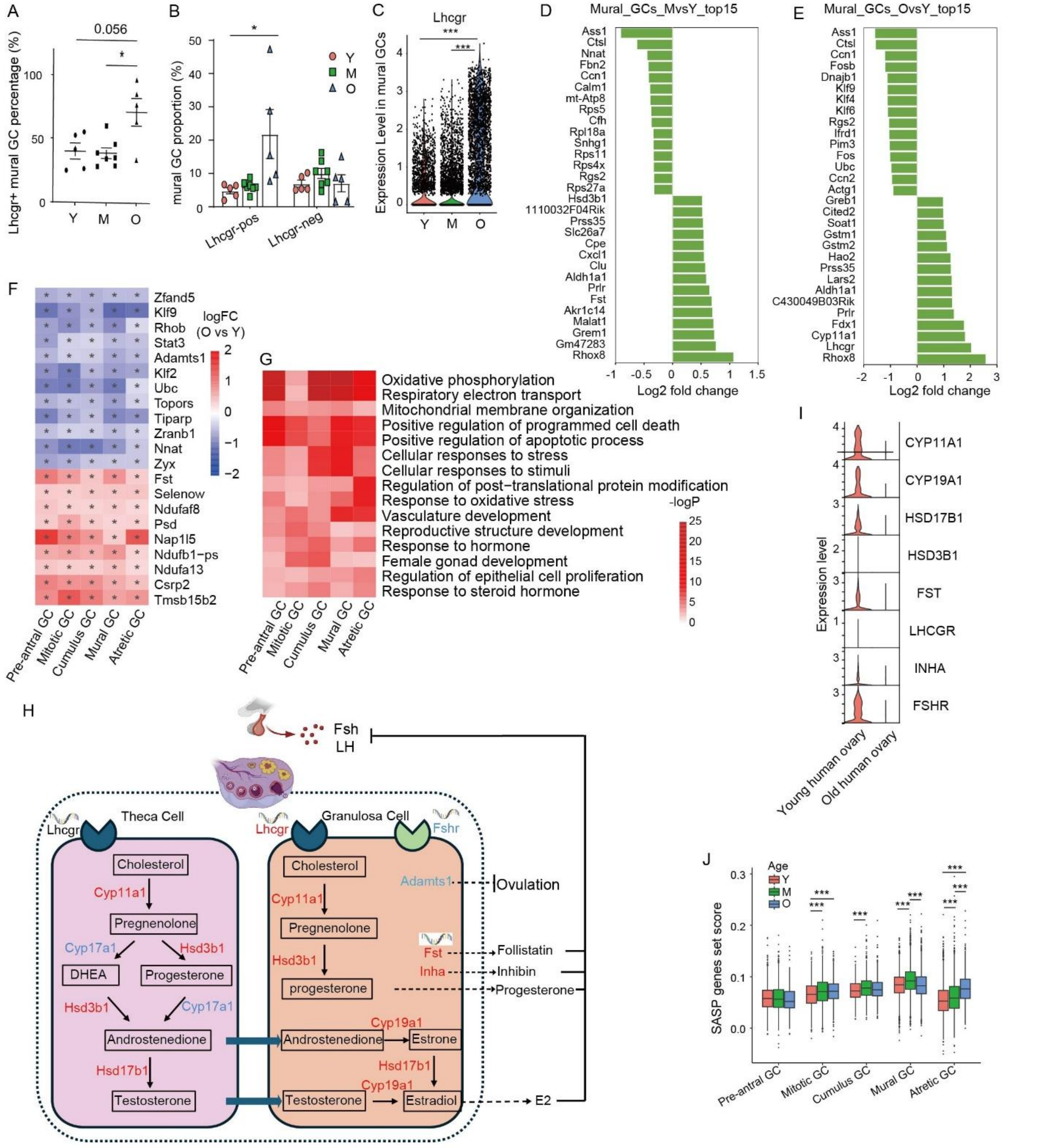

Fig. S7. Changes in granulosa cell subpopulations during mouse ovarian aging. (A) Scatter plots showing the percentage of *Lhcgr*$^{+}$ cells in mural granulosa cells (GC). **(B)** Bar plots showing the cell proportion of *Lhcgr*$^{+}$ and *Lhcgr*$^{-}$ mural GC in young (Y), peri-estropause (M), and post-estropause (O) ovaries. **(C)** Violin plots showing the expression level of *Lhcgr* in mural GC at each age group. **(D-E)** Bar plots showing the top 15 upregulated and downregulated DEGs in mural GC in peri-estropause ovaries (M vs Y, **D**) and post-estropause ovaries (O vs Y, **E**) compared with young ovaries. **(F)** Heatmap showing the $\log_2$ fold changes in gene expression (O vs Y) of

monotonic DEGs shared by all subpopulations of GC. * Indicates a statistically significant difference ($Padj < 0.05$). **(G)** Heatmap showing the representative GO terms of monotonic DEGs in each subpopulation of GC. **(H)** Diagram showing ovarian steroidogenesis by GC and theca cell (TC). Color of genes indicates upregulation (red) or downregulation (blue) of the gene expression in post-estropause (O) ovary compared to young (Y) ovary. **(I)** Violin plots showing the expression of hormone-related genes in human ovarian granulosa cells in young and aged ovaries. **(J)** Box plots showing the SASP gene set score in each subclusters of GC at each age group (Wilcoxon test, *** $Padj < 0.001$).

Figure S8

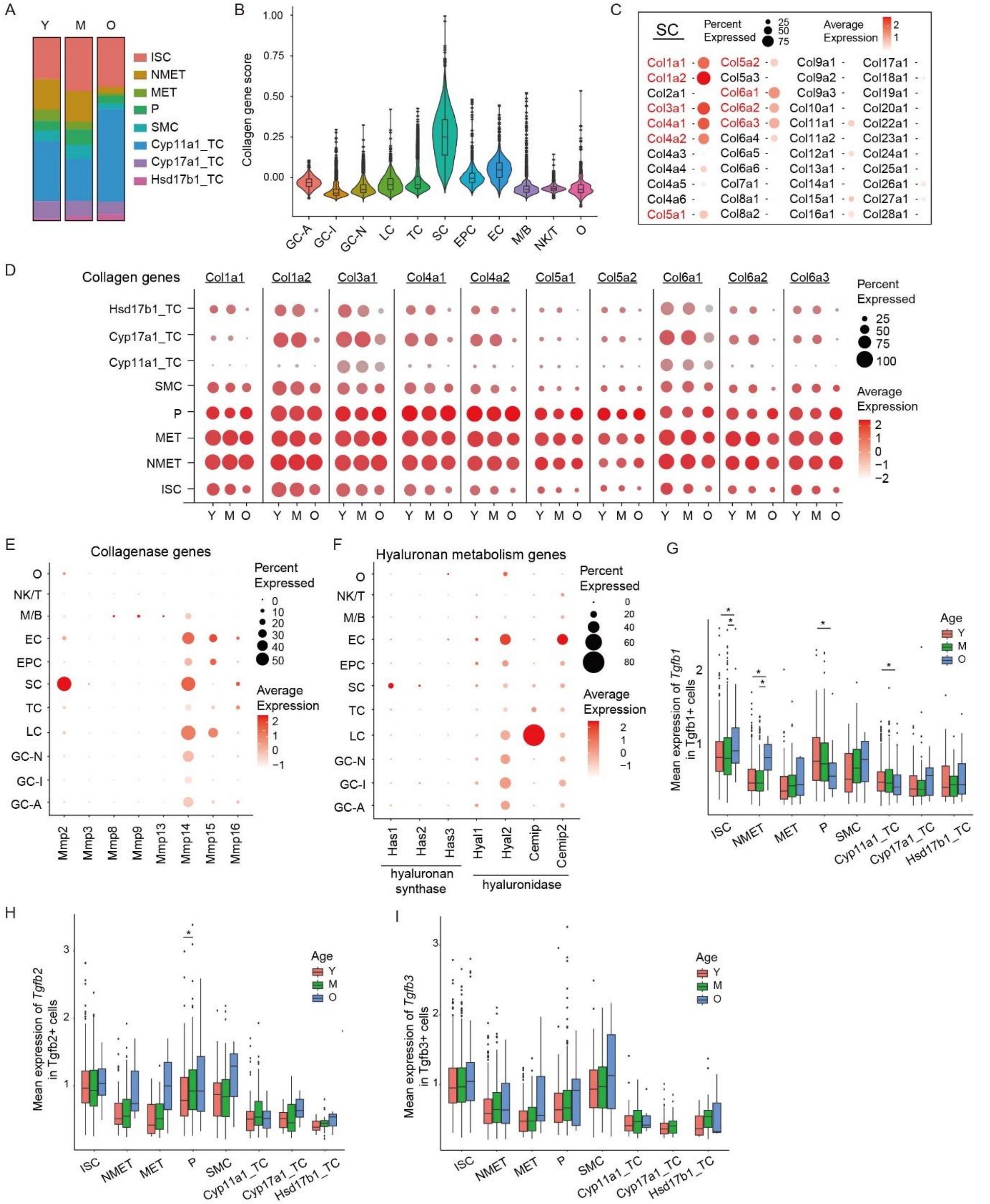

Fig. S8. Change in stromal and theca cell subpopulations during mouse ovarian aging. (A) Bar plots showing the distribution of subpopulations of SCTC at each age. **(B)** Violin and box plots showing the scores of collagen genes in each cell type of mouse ovarian cells. **(C)** Dot plots showing the expression of each collagen gene in stromal cells (SC). **(D)** Dot plot showing the expression of relatively highly expressed collagen genes in each stromal and theca cell (SCTC)

subtype at each age. ISC, interstitial stromal cells; SMC, smooth muscle cells; P, pericytes; MET, mitotic early theca cells; NMET, non-mitotic early theca cells. Cyp17a1-TC, *Cyp17a1*-high theca cells; Cyp11a1-TC, *Cyp11a1*-high theca cells; Hsd17b1-TC, *Hsd17b1*-high theca cells. **(E)** Dot plots showing the expression of collagenase genes in each cell type of mouse ovarian cells. **(F)** Dot plots showing the expression of hyaluronan metabolism-related genes in each cell type of mouse ovarian cells. **(G-I)** box plots showing the expression of *Tgfb1* (**G**), *Tgfb2* (**H**), and *Tgfb3* (**I**) in each SCTC subtype at each age. Wilcoxon test; * Padj < 0.05.

Figure S9

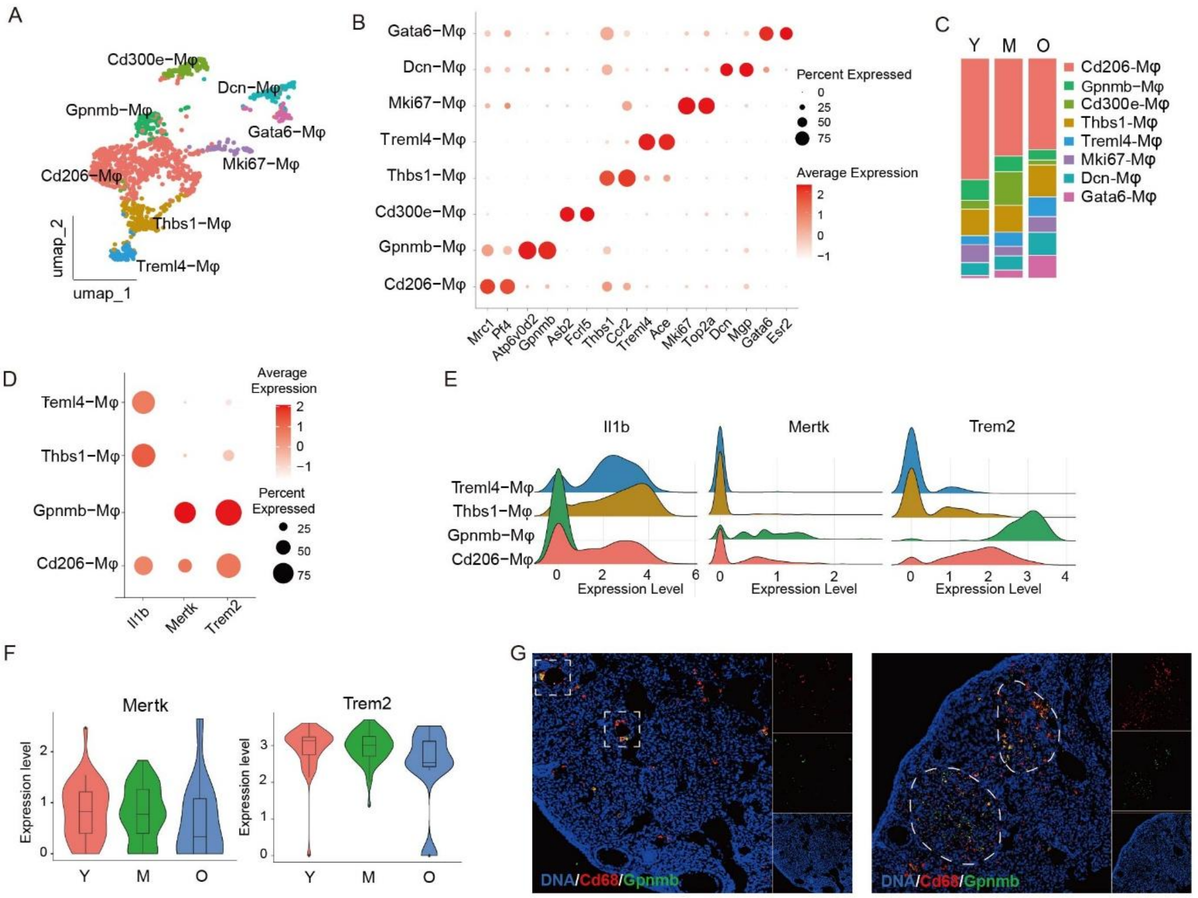

Fig. S9. Change in macrophage subpopulations during mouse ovarian aging. **(A)** UMAP plots showing the subpopulations of macrophages (Mφ). **(B)** Dot plots showing the expression of representative genes in subtypes of macrophage. **(C)** Proportion of different subpopulations of macrophage among macrophage clusters. **(D)** Dot plot showing the expression of inflammatory gene Il1b and endocytosis gene *Mertk* and *Trem2* in subcluster of macrophages. **(E)** Ridge Plot showing the expression of *Il1b*, *Mertk*, and *Trem2* in different macrophages. **(F)** Violin and box plot showing the expression of *Mertk* and *Trem2* in Atp6v0d2-Mφ at different age groups. **(G)** Representative images of atretic follicles showing *Gpnmb*-positive macrophages in small degenerated atretic follicles (rectangle) and corpus luteum (circle) in young ovaries.

Figure S10

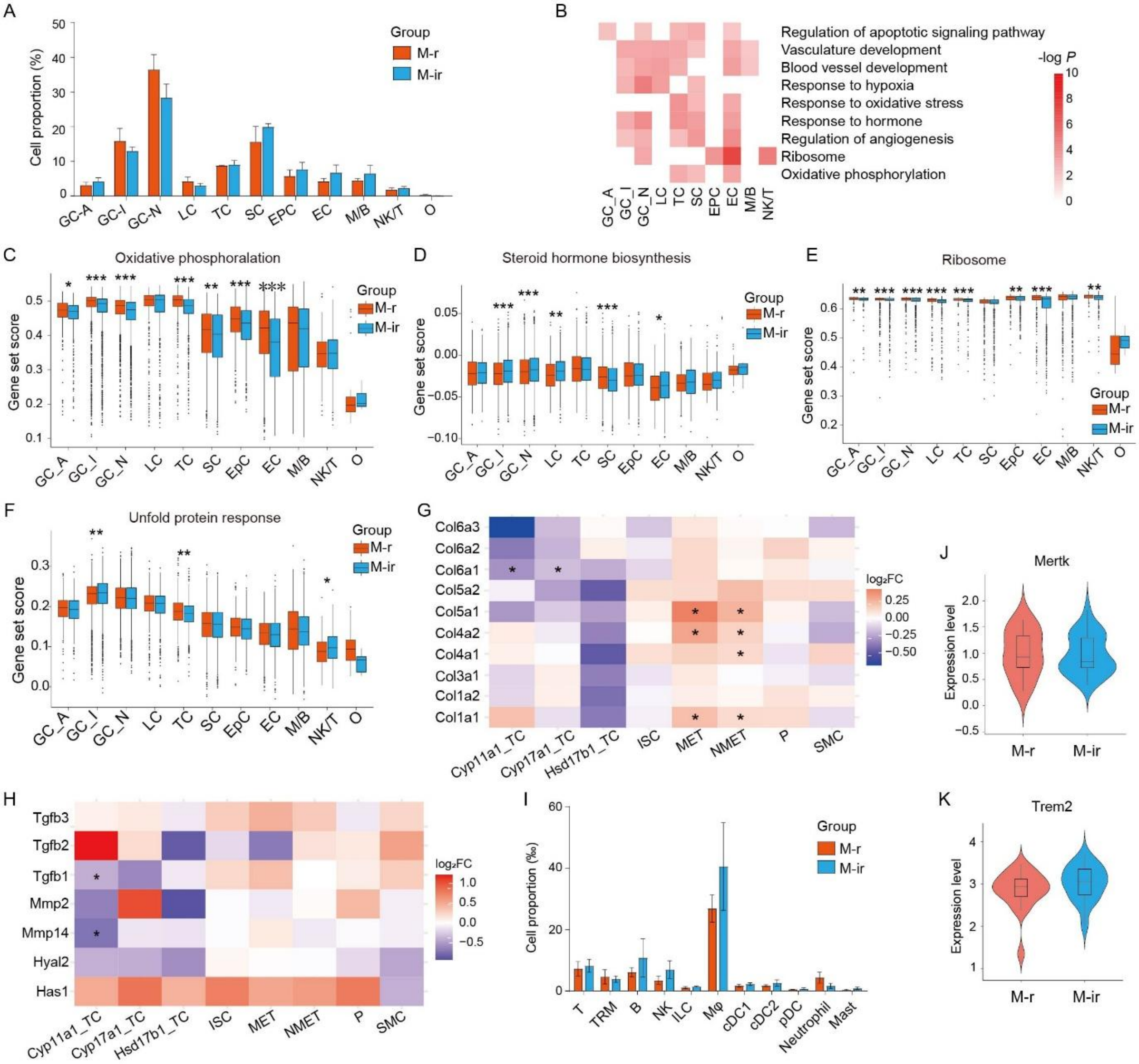

Fig. S10. Transcriptional dynamics of ovarian aging and senescent cell accumulation underlying the estropausal transition. (A) Bar plots showing the proportion of each cell type in peri-estropause ovaries with regular cycle (M-r) and irregular cycle (M-ir) (Mean ± SEM). **(B)** Heatmap showing the representative GO terms of DEGs between irregular cycling ovary and regular cycling ovary. **(C-F)** Box plots showing the gene set scores of oxidative phosphorylation **(C)** steroid hormone biosynthesis **(D)**, ribosome **(E)**, unfold protein response **(F)** in peri-estropause ovaries with regular cycle (M-r) and irregular cycle (M-ir) across all cell types. (Wilcoxon test; * Padj < 0.05, ** Padj < 0.01, *** Padj < 0.001). **(G)** Heatmap showing the $\log_2$fold change in expression of collagen genes in each stromal and theca cell (SCTC) subclusters, comparing irregular cycling ovaries to regular cycling counterparts at the peri-estropause stage (Wilcoxon test, * Padj < 0.05). **(H)** Heatmap showing the $\log_2$fold change in expression of collagenase genes, hyaluronan metabolism-related genes and *Tgfb* genes in subclusters of SCTC, comparing irregular cycling ovaries to regular cycling counterparts at the peri-estropause stage (Wilcoxon test, * Padj < 0.05). **(I)** Bar plots showing the proportion of each immune cell types in peri-estropause ovaries

with regular cycle (M-ir) and irregular cycle (M-ir) (Mean ± SEM). **(J-K)** Violin and box plot showing the expression of *Mertk* **(J)** and *Trem2* **(K)** in Atp6v0d2-Mφ in peri-estropause ovaries with regular cycle (M-ir) and irregular cycle (M-ir).

Figure S11

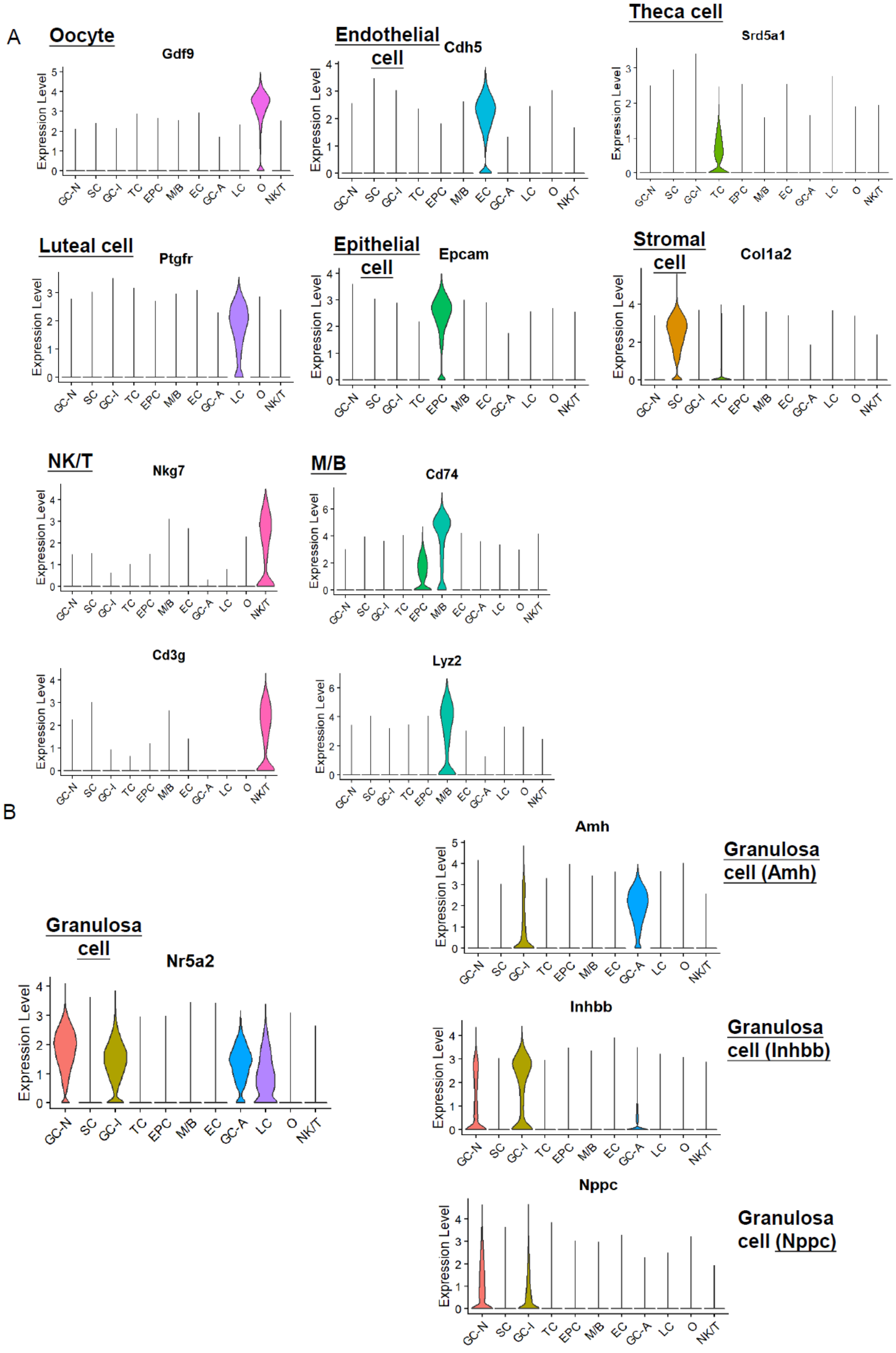

Fig. S11. Violin plots showing marker gene expression across cell clusters. (A) Cell types identified using well-established markers: oocyte (*Gdf9*), endothelial cell (*Cdh5*), stromal cell

(*Col1a2*, collagen gene), epithelial cell (*Epcam*), luteal cell (*Ptgfr*), theca cell (*Srd5a1*), monocyte/macrophage and B cell (M/B; *Cd74* for B cells, *Lyz2* for macrophages), and NK/T cell (*Nkg7* for NK cells, *Cd3g* for T cells). **(B)** Three granulosa cell clusters distinguished by expression of hormone-related genes: GC-A (*Amh*), GC-I (*Inhbb*), and GC-N (*Nppc*).

Figure S12

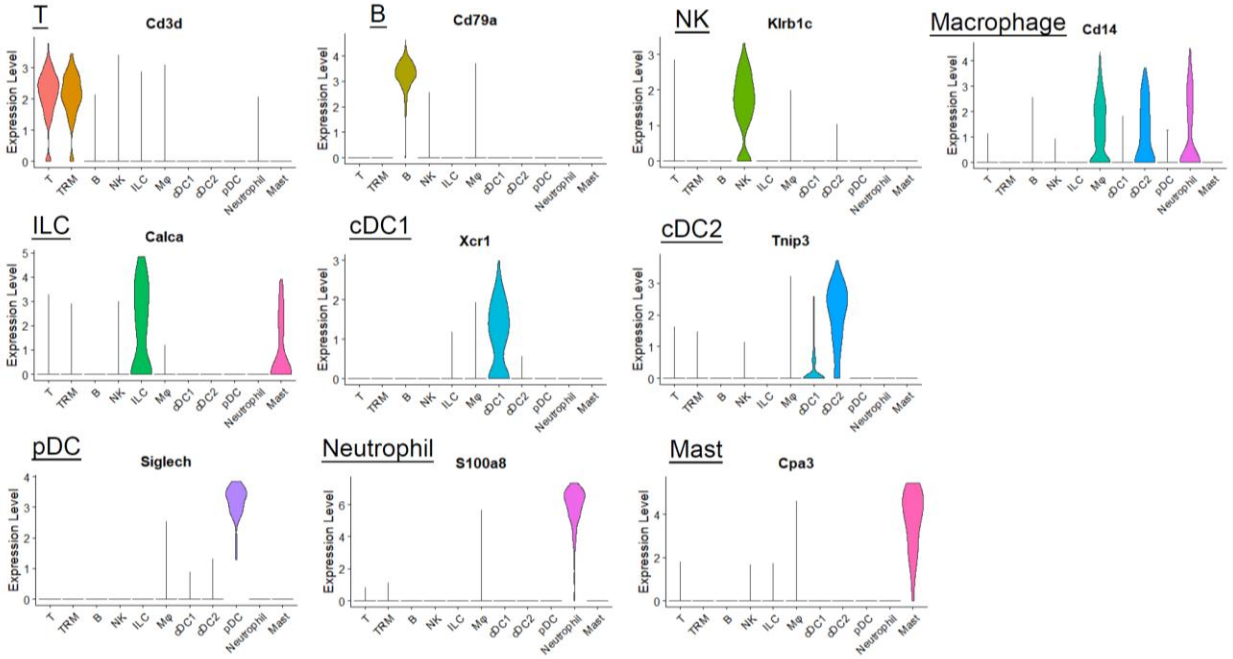

Fig. S12. Violin plots showing marker gene expression across cell clusters in the immune cell subpopulation. Markers include T cells (*Cd3d*), B cells (*Cd79a*), NK cells (*Klrb1c*), macrophages (*Cd14*), ILCs (*Calca*), cDC1 (*Xcr1*), cDC2 (*Tnip3*), pDC (*Siglech*), neutrophils (*S100a8*), and Mast (*Cpa3*).

Supplementary Table 1 Gene set lists

SASP	Unfolded protein response	Nuclear-encoded OXPHOS components	Mitochodia-encoded genes	Nfkb signaling pathway	DNA-repair	Collagen	M1 Pro-inflammation signature	M2 Anti-inflammation signature
Acvr1b	Abca7	Ndufa1	mt-Nd1	Abca1	Abl1	Col1a1	Ccl5	Ccl17
Ang	Abcb10	Ndufa2	mt-Nd2	Ackr3	Alkbh1	Col1a2	Ccl7	Ccl20
Angpt1	Agr2	Ndufa3	mt-Nd3	Areg	Apex1	Col2a1	Cd40	Ccl22
Angptl4	Amfr	Ndufa4	mt-Nd4	Atf3	Aptx	Col3a1	Cd86	Ccl24
Areg	Atf3	Ndufa5	mt-Nd4l	Atp2b1	Asf1a	Col4a1	Cxcl10	Ccl4
Axl	Atf4	Ndufa6	mt-Nd5	B4galt1	Atm	Col4a2	Cxcl11	Cd276
Bex3	Atf6	Ndufa7	mt-Nd6	B4galt5	Atr	Col4a3	Cxcl9	Ctsa
Bmp2	Atf6b	Ndufa8	mt-Cytb	Bcl2a1a	Atrx	Col4a4	Ido1	Ctsb
Bmp6	Bag3	Ndufa9	mt-Atp6	Bcl2a1b	Atxn3	Col4a5	Il1a	Ctsc
C3	Bak1	Ndufa10	mt-Atp8	Bcl2a1d	Blm	Col4a6	Il1b	Ctsd
Ccl1	Bax	Ndufa11	mt-Co1	Bcl3	Brca1	Col5a1	Il6	Egf
Ccl2	Bfar	Ndufb2	mt-Co2	Bcl6	Brca2	Col5a2	Irf1	Fasl
Ccl20	Bhlha15	Ndufb3	mt-Co3	Bhlhe40	Btg2	Col5a3	Irf5	Il4ra
Ccl24	Bok	Ndufb4		Birc2	Ccno	Col6a1	Kynu	Irf4
Ccl26	Casp12	Ndufb5		Birc3	Cdkn2d	Col6a2	Tnf	Lyve1
Ccl3	Ccnd1	Ndufb6		Bmp2	Cebpg	Col6a3	Il23a	Mmp14
Ccl4	Cdk5rap3	Ndufb7		Btg1	Cib1	Col6a4		Mmp19
Ccl5	Chac1	Ndufb8		Btg2	Csnk1d	Col6a5		Mmp9
Ccl7	Comp	Ndufb9		Btg3	Csnk1e	Col6a6		Msr1
Ccl8	Cops5	Ndufb10		Ccl12	Ddb1	Col7a1		Tgfb1
Cd55	Creb3	Ndufb11		Ccl20	Ddb2	Col8a1		Tgfb2
Cd9	Creb3l1	Ndufc1		Ccl4	Ercc1	Col8a2		Tgfb3
Csf1	Creb3l2	Ndufc2		Ccl5	Ercc2	Col9a1		Tnfsf12
Csf2	Creb3l3	Ndufs1		Ccnd1	Ercc3	Col9a2		Tnfsf8
Csf2rb	Creb3l4	Ndufs2		Ccnl1	Ercc4	Col9a3		Vegfa
Cst10	Crebrf	Ndufs3		Ccrl2	Ercc5	Col10a1		Vegfb
Ctnnb1	Dab2ip	Ndufs4		Cd44	Ercc6	Col11a1		Vegfc
Ctsb	Daxx	Ndufs5		Cd69	Ercc8	Col11a2		Vegfd
Cxcl1	Ddit3	Ndufs6		Cd80	Exo1	Col12a1		Vtcn1
Cxcl10	Ddrgk1	Ndufs7		Cd83	Fanca	Col13a1		Wnt7b
Cxcl12	Derl1	Ndufs8		Cdkn1a	Fancc	Col14a1		
Cxcl16	Derl2	Sdha		Cebpb	Fancg	Col15a1		
Cxcl2	Derl3	Sdhb		Cebpd	Fen1	Col16a1		
Cxcl3	Dnajb9	Sdhc		Cflar	Gadd45a	Col17a1		
Cxcr2	Dnajc3	Sdhd		Clcf1	Gadd45g	Col18a1		
Dkk1	Edem1	Uqcrc1		Csf1	Gtf2h1	Col19a1		
Edn1	Edem2	Uqcrc2		Csf2	Gtf2h4	Col20a1		
Egf	Edem3	Uqcrb		Cxcl10	Hmgb2	Col22a1		
Egfr	Eif2a	Uqcrq		Cxcl1	Hus1	Col23a1		
Ereg	Eif2ak2	Uqcrh		Cxcl2	Ighmbp2	Col24a1		
Esm1	Eif2ak3	Uqcr11		Cxcl5	Kat5	Col25a1		
Ets2	Eif2ak4	Uqcrfs1		Ddx58	Lig1	Col26a1		
Fas	Eif2s1	Uqcr10		Dennd5a	Lig3	Col27a1		
Fgf1	Ep300	Cox4l1		Dnajb4	Lig4	Col28a1		
Fgf2	Ermp1	Cox5a		Dram1	Mlh1			
Fgf7	Ern1	Cox5b		Dusp1	Mms19			
Gdf15	Ern2	Cox6a2		Dusp2	Mnat1			
Gem	Ero1a	Cox6a1		Dusp4	Mpg			

Gmfg	Erp27	Cox6b1		Dusp5	Msh2			
Hgf	Erp44	Cox6c		Edn1	Msh3			
Hmgb1	Faf2	Cox7a2		Efna1	Msh5			
Icam1	Fbxo6	Cox7a3		Egr1	Msh6			
Icam5	Ficd	Cox7a1		Egr2	Mutyh			
Igf1	Herpud1	Cox7a2l		Egr3	Nbn			
Igfbp1	Herpud2	Cox7b		Ehd1	Nhej1			
Igfbp2	Hsf1	Cox7c		Ets2	Nthl1			
Igfbp3	Hsp90aa1	Cox8a		F2rl1	Ogg1			
Igfbp4	Hspa1a	Ndufa4		F3	Parp1			
Igfbp5	Hspa1b	Cox17		Fjx1	Parp3			
Igfbp6	Hspa1l	Surf1		Fos	Pms1			
Igfbp7	Hspa2	Sco1		Fosb	Pms2			
Il10	Hspa4l	Cox11		Fosl1	Pnkp			
Il13	Hspa5	Cox15		Fosl2	Pola1			
Il15	Hspa8	Atp5a1		Fut4	Pold1			
Il18	Hspa9	Atp5b		G0s2	Pole			
Il1a	Hspa13	Atp5c1		Gadd45a	Pole2			
Il1b	Hspa14	Atp5d		Gadd45b	Polg			
Il2	Hspb1	Atp5e		Gch1	Polh			
Il6	Hspb8	Atp5j		Gem	Poli			
Il6st	Hspd1	Atp5o		Gfpt2	Poll			
Il7	Hsph1	Atp5f1		Gpr183	Polq			
Inha	Ifng	Atp5g1		Hbegf	Prkcg			
Iqgap2	Jkamp	Atp5g2		Hes1	Rad1			
Itga2	Manf	Atp5g3		Icam1	Rad17			
Itpka	Mfn2	Atp5h		Icosl	Rad21			
Jun	Nck1	Atp5i		Id2	Rad23a			
Kitl	Nck2	Atp5j2		Ier2	Rad23b			
Lcp1	Nfe2l2	Atp5l		Ier3	Rad50			
Mif	Optn	Atp5s		Ier5	Rad51			
Mmp13	Pacrg	Atpif1		Ifih1	Rad51b			
Mmp10	Parp16			Ifit2	Rad51c			
Mmp12	Pdia6			Ifngr2	Rad52			
Mmp14	Pik3r1			Il12b	Rad54b			
Mmp2	Pmp22			Il15ra	Rad54l			
Mmp3	Ppp1r15a			Il18	Rad9a			
Mmp9	Ptpn1			Il1a	Rbbp8			
Nap1l4	Ptpn2			Il1b	Recql			
Nrg1	Qrich1			Il23a	Recql4			
Pappa	Rhbdd1			Il6	Recql5			
Pecam1	Selenos			Il6st	Rev1			
Pgf	Serp1			Il7r	Rfc3			
Pigf	Serp2			Inhba	Rpa1			
Plat	Stc2			Irf1	Rpain			
Plau	Stt3b			Irs2	Ruvbl2			
Plaur	Stub1			Jag1	Setx			
Ptbp1	Syvn1			Jun	Smc1a			
Ptger2	Tbl2			Junb	Smug1			
Ptges	Thbs1			Kdm6b	Sod1			
Rps6ka5	Thbs4			Klf10	Sumo1			
Scamp4	Tm7sf3			Klf2	Tdg			
Selplg	Tmbim6			Klf4	Gm5806			
Sema3f	Tmed2			Klf6	Trp53			

Serpinb3a	Tmem33			Klf9	Trp73			
Serpine1	Tmem129			Kynu	Trex2			
Serpine2	Tmtc4			Lamb3	Ube2a			
Spp1	Ube2j2			Ldlr	Ube2b			
Spx	Ubxn4			Lif	Ube2n			
Timp2	Ufl1			Litaf	Ube2v1			
Tnf	Umod			Maff	Ube2v2			
Tnfrsf11b	Upf2			Map2k3	Ung			
Tnfrsf1a	Upf3a			Map3k8	Upf1			
Tnfrsf1b	Upf3b			Mcl1	Uvrag			
Tubgcp2	Vapb			Msc	Vcp			
Vegfa	Wfs1			Mxd1	Wrnip1			
Vegfc	Xbp1			Myc	Xab2			
Vgf	Yod1			Nampt	Xpc			
Wnt16	Creb3			Nfat5	Xrcc2			
Wnt2	Ep300			Nfe2l2	Xrcc3			
Ngf	Xbp1			Nfil3	Xrcc4			
Timp1	Abca7			Nfkb1	Xrcc6			
Inhba	Agr2			Nfkb2				
Tgfb1	Amfr			Nfkbia				
Cxcl5	Atf3			Nfkbie				
Igf2r	Atf4			Ninj1				
Serpinb2	Atf6			Nr4a1				
Csf3	Atf6b			Nr4a2				
Iqgap1	Bak1			Nr4a3				
	Bax			Olr1				
	Bfar			Panx1				
	Bhlha15			Pde4b				
	Bok			Pdlim5				
	Casp12			Pfkfb3				
	Ccnd1			Phlda1				
	Cdk5rap3			Phlda2				
	Cops5			Plau				
	Creb3			Plaur				
	Creb3l1			Plek				
	Creb3l2			Plk2				
	Creb3l3			Pmepa1				
	Creb3l4			Pnrc1				
	Crebrf			Ppp1r15a				
	Ddit3			Ptger4				
	Ddrgk1			Ptgs2				
	Derl1			Ptpre				
	Derl2			Ptx3				
	Derl3			Rcan1				
	Dnajc3			Rel				
	Eif2a			Rela				
	Eif2ak2			Relb				
	Eif2ak3			Rhob				
	Eif2ak4			Ripk2				
	Eif2s1			Rnf19b				
	Ep300			Sat1				
	Ermp1			Sdc4				
	Ern1			Serpinb2				
	Ern2			Serpinb8				

	Ero1a			Serpine1				
	Ficd			Sgk1				
	Herpud1			Slc16a6				
	Herpud2			Slc2a3				
	Hspa5			Slc2a6				
	Ifng			Smad3				
	Nck1			Snn				
	Nck2			Socs3				
	Nfe2l2			Sod2				
	Parp16			Sphk1				
	Pdia6			Spsb1				
	Pik3r1			Sqstm1				
	Ppp1r15a			Stat5a				
	Ptpn1			Tank				
	Ptpn2			Tap1				
	Qrich1			Tgif1				
	Selenos			Tiparp				
	Serp1			Tlr2				
	Serp2			Tnc				
	Stc2			Tnf				
	Stub1			Tnfaip2				
	Tbl2			Tnfaip3				
	Tmed2			Tnfaip6				
	Tmem33			Tnfaip8				
	Tmtc4			Tnfrsf9				
	Ufl1			Tnip1				
	Vapb			Tnip2				
	Wfs1			Traf1				
	Xbp1			Trib1				
	Yod1			Trip10				
	Abcb10			Tsc22d1				
	Hspd1			Tubb2a				
	Abca7			Vegfa				
	Abcb10			Yrdc				
	Agr2			Zbtb10				
	Amfr			Zc3h12a				
	Atf3			Zfp36				
	Atf4							
	Atf6							
	Atf6b							
	Atxn3							
	Aup1							
	Bag3							
	Bag6							
	Bak1							
	Bax							
	Bfar							
	Bhlha15							
	Bok							
	Casp12							
	Ccnd1							
	Cdk5rap3							
	Cops5							
	Creb3							

	Creb3l1							
	Creb3l2							
	Creb3l3							
	Creb3l4							
	Crebrf							
	Cul3							
	Daxx							
	Ddit3							
	Ddrgk1							
	Derl1							
	Derl2							
	Derl3							
	Dnajb12							
	Dnajb14							
	Dnajc3							
	Dnajc18							
	Eif2a							
	Eif2ak2							
	Eif2ak3							
	Eif2ak4							
	Eif2s1							
	Ep300							
	Ermp1							
	Ern1							
	Ern2							
	Ero1a							
	Ficd							
	Hdac6							
	Herpud1							
	Herpud2							
	Hsf1							
	Hspa1a							
	Hspa1b							
	Hspa1l							
	Hspa2							
	Hspa5							
	Hspa8							
	Hspa9							
	Hspa13							
	Hspa14							
	Hspb8							
	Hspd1							
	Ifng							
	Klhl15							
	Nck1							
	Nck2							
	Nfe2l2							
	Ngly1							
	Optn							
	Pacrg							
	Parp16							
	Pdia6							
	Pik3r1							
	Pmp22							

	Ppp1r15a							
	Ptpn1							
	Ptpn2							
	Qrich1							
	Rhbdd1							
	Rnf5							
	Rnf126							
	Rnf185							
	Sdf2l1							
	Selenos							
	Serp1							
	Serp2							
	Stc2							
	Stub1							
	Tbl2							
	Tm7sf3							
	Tmbim6							
	Tmed2							
	Tmem33							
	Tmtc4							
	Tor1a							
	Ube2w							
	Ufd1							
	Ufl1							
	Uggt1							
	Uggt2							
	Umod							
	Vapb							
	Vcp							
	Wfs1							
	Xbp1							
	Yod1							
	Abca7							
	Abcb10							
	Agr2							
	Amfr							
	Atf3							
	Atf4							
	Atf6							
	Atf6b							
	Atxn3							
	Aup1							
	Bag3							
	Bag6							
	Bak1							
	Bax							
	Bfar							
	Bhlha15							
	Bok							
	Casp12							
	Ccnd1							
	Cdk5rap3							
	Chac1							
	Clu							

	Comp							
	Cops5							
	Creb3							
	Creb3l1							
	Creb3l2							
	Creb3l3							
	Creb3l4							
	Crebrf							
	Cul3							
	Dab2ip							
	Daxx							
	Ddit3							
	Ddrgk1							
	Derl1							
	Derl2							
	Derl3							
	Dnajb9							
	Dnajb12							
	Dnajb14							
	Dnajc3							
	Dnajc18							
	Edem1							
	Edem2							
	Edem3							
	Eif2a							
	Eif2ak2							
	Eif2ak3							
	Eif2ak4							
	Eif2s1							
	Ep300							
	Ermp1							
	Ern1							
	Ern2							
	Ero1a							
	Erp27							
	Erp44							
	F12							
	Faf2							
	Fbxo6							
	Ficd							
	Hdac6							
	Herpud1							
	Herpud2							
	Hsf1							
	Hsp90aa1							
	Hspa1a							
	Hspa1b							
	Hspa1l							
	Hspa2							
	Hspa4l							
	Hspa5							
	Hspa8							
	Hspa9							
	Hspa13							

	Hspa14							
	Hspb1							
	Hspb8							
	Hspd1							
	Hsph1							
	Ifng							
	Jkamp							
	Klhl15							
	Manf							
	Mfn2							
	Nck1							
	Nck2							
	Nfe2l2							
	Ngly1							
	Optn							
	Pacrg							
	Parp16							
	Pdia6							
	Pik3r1							
	Pmp22							
	Ppp1r15a							
	Ptpn1							
	Ptpn2							
	Qrich1							
	Rhbdd1							
	Rnf5							
	Rnf126							
	Rnf185							
	Sdf2l1							
	Selenos							
	Serp1							
	Serp2							
	Stc2							
	Stt3b							
	Stub1							
	Syvn1							
	Tbl2							
	Thbs1							
	Thbs4							
	Tm7sf3							
	Tmbim6							
	Tmed2							
	Tmem33							
	Tmem129							
	Tmtc4							
	Tor1a							
	Ube2j2							
	Ube2w							
	Ubxn4							
	Ufd1							
	Ufl1							
	Uggt1							
	Uggt2							
	Umod							

	Upf2							
	Upf3a							
	Upf3b							
	Vapb							
	Vcp							
	Wfs1							
	Xbp1							
	Yod1							
	Abca7							
	Abcb10							
	Agr2							
	Amfr							
	Atf3							
	Atf4							
	Atf6							
	Atf6b							
	Atxn3							
	Aup1							
	Bag3							
	Bag6							
	Bak1							
	Bax							
	Bfar							
	Bhlha15							
	Bok							
	Casp12							
	Ccnd1							
	Cdk5rap3							
	Cops5							
	Creb3							
	Creb3l1							
	Creb3l2							
	Creb3l3							
	Creb3l4							
	Crebrf							
	Cul3							
	Daxx							
	Ddit3							
	Ddrgk1							
	Derl1							
	Derl2							
	Derl3							
	Dnajb12							
	Dnajb14							
	Dnajc3							
	Dnajc18							
	Eif2a							
	Eif2ak2							
	Eif2ak3							
	Eif2ak4							
	Eif2s1							
	Ep300							
	Ermp1							
	Ern1							

	Ern2							
	Ero1a							
	Ficd							
	Hdac6							
	Herpud1							
	Herpud2							
	Hsf1							
	Hspa1a							
	Hspa1b							
	Hspa1l							
	Hspa2							
	Hspa5							
	Hspa8							
	Hspa9							
	Hspa13							
	Hspa14							
	Hspb8							
	Hspd1							
	Ifng							
	Klhl15							
	Nck1							
	Nck2							
	Nfe2l2							
	Ngly1							
	Optn							
	Pacrg							
	Parp16							
	Pdia6							
	Pik3r1							
	Pmp22							
	Ppp1r15a							
	Ptpn1							
	Ptpn2							
	Qrich1							
	Rhbdd1							
	Rnf5							
	Rnf126							
	Rnf185							
	Sdf2l1							
	Selenos							
	Serp1							
	Serp2							
	Stc2							
	Stub1							
	Tbl2							
	Tm7sf3							
	Tmbim6							
	Tmed2							
	Tmem33							
	Tmtc4							
	Tor1a							
	Ube2w							
	Ufd1							
	Ufl1							

	Uggt1							
	Uggt2							
	Umod							
	Vapb							
	Vcp							
	Wfs1							
	Xbp1							
	Yod1							
	Agr2							
	Atf6							
	Bak1							
	Bax							
	Bok							
	Ern1							
	Pik3r1							
	Ptpn2							
	Tmem33							
	Xbp1							
	Atf6							
	Agr2							
	Tmem33							
	Agr2							
	Bok							
	Ptpn2							
	Tmem33							
	4930503B20Rik							
	Ahsa1							
	Ahsa2							
	Ahsp							
	Aip							
	Arl2							
	AU015836							
	B2m							
	Bag1							
	Bag5							
	Calr							
	Calr3							
	Calr4							
	Canx							
	Cct2							
	Cct3							
	Cct4							
	Cct5							
	Cct6a							
	Cct6b							
	Cct7							
	Cct8							
	Cct8l1							
	Cd74							
	Cdc37							
	Cdc37l1							
	Chchd4							
	Chordc1							
	Clgn							

	Clpx							
	Clu							
	Cryaa							
	Cryab							
	Cwc27							
	Dffa							
	Dnaja1							
	Dnaja2							
	Dnaja3							
	Dnaja4							
	Dnajb1							
	Dnajb2							
	Dnajb3							
	Dnajb4							
	Dnajb5							
	Dnajb6							
	Dnajb7							
	Dnajb8							
	Dnajb11							
	Dnajb12							
	Dnajb13							
	Dnajb14							
	Dnajc1							
	Dnajc3							
	Dnajc5							
	Dnajc7							
	Dnajc10							
	Dnajc18							
	Dnajc25							
	Dnlz							
	Entpd5							
	Ero1a							
	Ero1b							
	Erp27							
	Erp44							
	Fkbp1a							
	Fkbp1b							
	Fkbp4							
	Fkbp5							
	Fkbp6							
	Fkbp8							
	Fkbp9							
	Grn							
	Grpel1							
	Grpel2							
	H2-DMa							
	H2-DMb1							
	H2-DMb2							
	Hsp90aa1							
	Hsp90ab1							
	Hsp90b1							
	Hspa1a							
	Hspa1b							
	Hspa1l							

	Hspa2							
	Hspa4l							
	Hspa5							
	Hspa8							
	Hspa9							
	Hspa13							
	Hspa14							
	Hspb1							
	Hspb6							
	Hspd1							
	Hspe1							
	Hsph1							
	Mesd							
	Mkks							
	Nktr							
	Nppa							
	Nppb							
	Nppc							
	Nudc							
	Nudcd2							
	Nudcd3							
	P3h1							
	P4hb							
	Pdcd5							
	Pdcl							
	Pdcl3							
	Pdia2							
	Pdia3							
	Pdia4							
	Pdia5							
	Pdilt							
	Pdrg1							
	Pex19							
	Pfdn1							
	Pfdn2							
	Pfdn4							
	Pfdn5							
	Pfdn6							
	Pofut2							
	Ppia							
	Ppib							
	Ppic							
	Ppid							
	Ppie							
	Ppif							
	Ppig							
	Ppih							
	Ppil1							
	Ppil2							
	Ppil3							
	Ppil6							
	Prdx4							
	Ptges3							
	Ptges3l							

	Qsox1							
	Qsox2							
	Ranbp2							
	Sdf2							
	Sdf2l1							
	Selenof							
	Sgta							
	Sgtb							
	Sh3glb1							
	St13							
	Stub1							
	Tbca							
	Tbcc							
	Tbcd							
	Tbce							
	Tbcel							
	Tcp1							
	Tor1a							
	Tor1b							
	Tor2a							
	Trap1							
	Ube4b							
	Umod							
	Unc45a							
	Unc45b							
	Vbp1							
	Wdr83os							
	Zmynd10							
	B2m							
	Bag5							
	Dnaja2							
	Dnaja4							
	Dnajb2							
	Hsp90aa1							
	Hspa1a							
	Hspa1b							
	Hspa1l							
	Hspa2							
	Hspa5							
	Hspa8							
	Hspa9							
	Hspa13							
	Hspa14							
	Hspd1							
	Pdcl							
	St13							
	Amfr							
	Atf4							
	Atf6							
	Atf6b							
	Atxn3							
	Bag1							
	Bag2							
	Bak1							

	Bax							
	Bcap31							
	Bcl2							
	Calr							
	Canx							
	Capn1							
	Capn2							
	Ckap4							
	Cryaa							
	Cryab							
	Cul1							
	Dad1							
	Ddit3							
	Ddost							
	Derl1							
	Derl2							
	Derl3							
	Dnaja1							
	Dnaja2							
	Dnajb1							
	Dnajb11							
	Dnajb12							
	Dnajb2							
	Dnajc1							
	Dnajc10							
	Dnajc3							
	Dnajc5							
	Dnajc5b							
	Dnajc5g							
	Edem1							
	Edem2							
	Edem3							
	Eif2ak1							
	Eif2ak2							
	Eif2ak3							
	Eif2ak4							
	Eif2s1							
	Erlec1							
	Ern1							
	Erp29							
	Fbxo2							
	Fbxo6							
	Ganab							
	Herpud1							
	Hsp90aa1							
	Hsp90ab1							
	Hsp90b1							
	Hspa1a							
	Hspa1a							
	Hspa1l							
	Hspa2							
	Hspa4l							
	Hspa5							
	Hspa8							

	Hspbp1							
	Hsph1							
	Hyou1							
	Lman1							
	Lman1l							
	Lman2							
	Man1a							
	Man1a2							
	Man1b1							
	Man1c1							
	Map2k7							
	Map3k5							
	Mapk10							
	Mapk8							
	Mapk9							
	Mbtps1							
	Mbtps2							
	Mogs							
	Nfe2l2							
	Ngly1							
	Nploc4							
	Nsfl1c							
	Os9							
	P4hb							
	Pdia3							
	Pdia4							
	Pdia6							
	Plaa							
	Ppp1r15a							
	Preb							
	Prkcsh							
	Rad23a							
	Rad23b							
	Rnf185							
	Rnf5							
	Rpn1							
	Rpn2							
	Sar1a							
	Sar1b							
	Sec13							
	Sec23a							
	Sec23b							
	Sec24a							
	Sec24b							
	Sec24c							
	Sec24d							
	Sec31a							
	Sec31b							
	Sec61a1							
	Sec61a2							
	Sec61b							
	Sec61g							
	Sec62							
	Sec63							

	Sel1l							
	Sil1							
	Skp1a							
	Ssr1							
	Ssr2							
	Ssr3							
	Ssr4							
	Stt3a							
	Stt3b							
	Stub1							
	Svip							
	Syvn1							
	Traf2							
	Tram1							
	Tram1l1							
	Tusc3							
	Txndc5							
	Ube2d1							
	Ube2d2a							
	Ube2d2b							
	Ube2d3							
	Ube2g1							
	Ube2g2							
	Ube2j1							
	Ube2j2							
	Ube4b							
	Ubqln1							
	Ubqln2							
	Ubqln3							
	Ubqln4							
	Ubxn1							
	Ubxn2a							
	Ubxn4							
	Ubxn6							
	Ubxn8							
	Uggt1							
	Uggt2							
	Vcp							
	Wfs1							
	Xbp1							
	Yod1							
